# Traditional Medicinal Uses, Phytoconstituents, Bioactivities, and Toxicities of *Erythrina abyssinica* Lam. ex DC. (Fabaceae): A Systematic Review

**DOI:** 10.1155/2021/5513484

**Published:** 2021-03-03

**Authors:** Samuel Baker Obakiro, Ambrose Kiprop, Elizabeth Kigondu, Isaac K'Owino, Mark Peter Odero, Scolastica Manyim, Timothy Omara, Jane Namukobe, Richard Oriko Owor, Yahaya Gavamukulya, Lydia Bunalema

**Affiliations:** ^1^Department of Pharmacology and Therapeutics, Faculty of Health Sciences, Busitema University, P.O. Box 1460, Mbale, Uganda; ^2^Department of Chemistry and Biochemistry, School of Sciences and Aerospace Studies, Moi University, P.O. Box 3900-30100, Eldoret, Kenya; ^4^Centre of Traditional Medicine and Drug Research, Kenya Medical Research Institute, P.O. Box 54840-00200, Nairobi, Kenya; ^10^Department of Pharmacology and Therapeutics, School of Biomedical Sciences, Makerere University College of Health Sciences, P.O. Box 7062, Kampala, Uganda; ^5^Department of Pure and Applied Chemistry, Faculty of Science, Masinde-Muliro University, P.O. Box 190-50100, Kakamega, Kenya; ^3^Africa Centre of Excellence II in Phytochemicals, Textiles and Renewable Energy (ACE II PTRE), Moi University, P.O. Box 3900-30100, Eldoret, Kenya; ^9^Department of Biochemistry and Molecular Biology, Faculty of Health Sciences, Busitema University, P.O. Box 1460, Mbale, Uganda; ^7^Department of Chemistry, School of Physical Sciences, College of Natural Sciences, Makerere University, P.O. Box 7062, Kampala, Uganda; ^6^Department of Quality Control and Quality Assurance, Product Development Directory, AgroWays Uganda Limited, Plot 34-60, Kyabazinga Way, P.O. Box 1924, Jinja, Uganda; ^8^Department of Chemistry, Faculty of Science Education, Busitema University, P.O. Box 236, Tororo, Uganda

## Abstract

**Background:**

Many studies have been undertaken on the medicinal values of *Erythrina abyssinica* Lam. ex DC. (Fabaceae). The details, however, are highly fragmented in different journals, libraries, and other publication media. This study was therefore conducted to provide a comprehensive report on its ethnobotany, ethnomedicinal uses, phytochemicals, and the available pharmacological evidence supporting its efficacy and safety in traditional medicine.

**Method:**

We collected data using a PROSPERO registered systematic review protocol on the ethnobotany, phytochemistry, and ethnopharmacology of *Erythrina abyssinica* from 132 reports that were retrieved from electronic databases. Documented local names, morphology, growth habit and habitat, ethnomedicinal and nonmedicinal uses, diseases treated, parts used, method of preparation and administration, extraction and chemical identity of isolated compounds, and efficacy and toxicity of extracts and isolated compounds were captured. Numerical data were summarized into means, percentages, and frequencies and presented as graphs and tables.

**Results:**

*Erythrina abyssinica* is harvested by traditional herbal medicine practitioners in East, Central, and South African communities to prepare herbal remedies for various human and livestock ailments. These include bacterial and fungal infections, tuberculosis, malaria, HIV/AIDS, diarrhea, cancer, meningitis, inflammatory diseases, urinary tract infections, wounds, diabetes mellitus, and skin and soft tissue injuries. Different extracts and phytochemicals from parts of *E. abyssinica* have been scientifically proven to possess anti-inflammatory, antibacterial, antioxidant, antiplasmodial, antiproliferative, antifungal, antimycobacterial, antidiarrheal, anti-HIV 1, antidiabetic, and antiobesity activities. This versatile pharmacological activity is due to the abundant flavonoids, alkaloids, and terpenoids present in its different parts.

**Conclusion:**

*Erythrina abyssinica* is an important ethnomedicinal plant in Africa harboring useful pharmacologically active phytochemicals against various diseases with significant efficacies and minimal toxicity to mammalian cells. Therefore, this plant should be conserved and its potential to provide novel molecules against diseases be explored further. Clinical trials that evaluate the efficacy and safety of extracts and isolated compounds from *E. abyssinica* are recommended.

## 1. Introduction

*Erythrina abyssinica* Lam. ex DC. (Fabaceae) is an important medicinal plant as evidenced by the existence of its names in various local languages and high frequency of citation in ethnobotanical surveys [1–4]. The genus *Erythrina* derives from the Greek word “*erythros*,” translated to mean red (a reflection of the showy red flowers of its various species). The epithet ‘‘*abyssinica*” means ‘‘from Ethiopia” [5]. The *Erythrina* genus houses at least 120 species distributed mainly in tropical and subtropical zones [6]. Plants in this genus are usually referred to as “coral trees” due to their red flowers and branches that resemble the shape of sea coral [7]. *Erythrina abyssinica* is a deciduous leguminous tree native to East Africa but also found in Central and South Africa [8, 9]. Tropical Asia and Central America have *E. abyssinica* as an exotic species. The common English names of *E. abyssinica* are coral tree, Uganda coral, kaffir boom, erythrina, flame tree, red-hot-poker tree, and lucky-bean tree [10]. Some of the local names used across indigenous communities are summarized in Table 1.

Medicinal plants have been a veritable source of cure for a number of human and livestock diseases, and thus, they are widely used in many communities. This is because plants house abundant secondary metabolites (phytochemicals) with potential pharmacological activities. These include flavonoids, alkaloids, terpenoids, phenols, chalcones, quinones, aromatic hydrocarbons, chromones, and coumarins. It is these phytochemicals that are locally extracted in herbal preparations and used as remedies for the management of several diseases. The World Health Organization (WHO) estimated that 80% of the world's population especially in low- and middle-income countries rely on herbal medicines for primary health care [30]. The use of herbal medicines in the management of several ailments among people continues to gain momentum due to their availability, affordability, perceived effectiveness, and cultural acceptability across ethnic backgrounds [31].

Globally, there has been an increase in natural product research in the last two decades [30, 32]. This has been partly in response to the increasing antimicrobial resistance, emergence of new diseases, and decrease in the chemical diversity of natural product libraries [30, 32–36]. It has also been so in an effort to continue the search for more effective, safer, and cheaper therapeutic agents for existing diseases, to substitute expensive prescription drugs [37–40]. *Erythrina abyssinica* is among those revered plants [40, 41] that has been widely researched [3]. However, the information on it is highly fragmented in different journals, books, university libraries, and other publication media platforms. This review was therefore undertaken to compile a comprehensive document that describes the ethnobotany, phytochemistry, and ethnopharmacology of *E. abyssinica* so as to generate integrated and sufficient scientific evidence to support its medicinal use. The study further emphasizes the importance of conserving this medicinal plant amidst the growing destruction of natural resources for settlement, industrialization, construction, and energy production [27, 42–47].

## 2. Methods

### 2.1. Protocol Registration and Reporting

**Figure 1:**
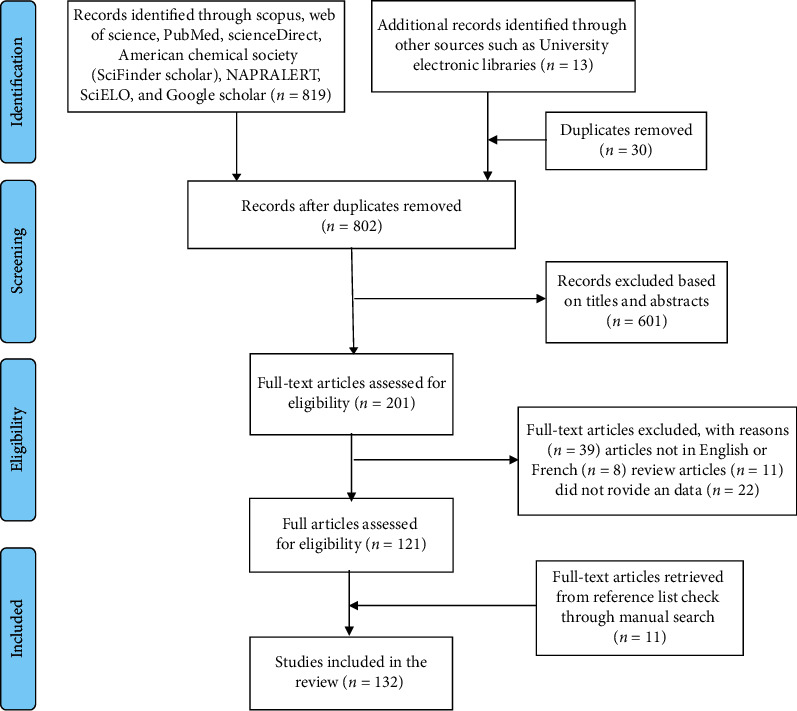
PRISMA flow diagram showing the search and retrieval steps of the study (adopted from Moher et al. [48]).

The protocol used in this systematic review was registered with the International Prospective Register of Systematic Reviews (PROSPERO) and can be accessed from their website (https://www.crd.york.ac.uk/prospero/display_record.php?ID=CRD42020187081) with the registration number CRD42020187081. The Preferred Reporting Items for the Systematic Reviews and Meta-Analyses (PRISMA) guidelines [48] have been used in the reporting of this study (Figure 1).

### 2.2. Literature Search

Electronic data on ethnobotany, phytochemistry, efficacy, and toxicity of *E. abyssinica* were retrieved from electronic databases such as Scopus, Web of Science Core Collection, PubMed, American Chemical Society, ScienceDirect, Scientific Electronic Library Online (SciELO), Google Scholar, and NAPRALERT (a comprehensive natural products database with ethnomedical and pharmacological information of extracts and isolated compounds). Sets of keywords such as “ethnobotany,” “traditional medicine,” “ethnobotany,” “alternative medicine,” “ethnopharmacology,” “phytochemistry,” “extraction,” “isolation,” “efficacy,” “safety,” “toxicity,” “phytochemicals,” “structural elucidation,” and clinical study were combined with “*Erythrina abyssinica*.” The retrieved articles were downloaded and stored in EndNote X9 (Thomson Reuters, San Francisco, CA, USA) by three independent authors (SBO, TO, and YG). Duplicate articles were then removed from the file. Further, manual search from the reference lists of screened eligible articles and deposited electronic copies of dissertations and theses in University online libraries were done. The authors continuously received notifications of any new “similar reports” meeting the search criteria from ScienceDirect, Scopus, and Google Scholar.

### 2.3. Screening

Retrieved articles were first screened based on the titles and abstracts for relevance to the study by three independent reviewers (MPO, SM, and YG). Articles that reported on other species of *Erythrina* but not *abyssinica* and also *abyssinica* but not of genus *Erythrina* were also excluded. For example, we excluded articles on *Entada abyssinica, Erythrina variageta*, *Erythrina suberosa*, *Albuca abyssinica*, *Dregea abyssinica*, *Harrisonia abyssinica*, and *Wahlenbergia abyssinica* although they appeared in the search results. During the screening, every time a disagreement occurred it was resolved through a discussion between the reviewers and/or by the principal investigator (SBO). The eligible articles were then assessed further for inclusion in the study using the inclusion/exclusion criteria.

### 2.4. Inclusion and Exclusion Criteria

Full-text articles that at least reported on ethnobotany, ethnopharmacology, and phytochemistry of *Erythrina abyssinica* written in English or French but translated to English and published in peer-reviewed journals, reports, books, theses, and dissertations dated until January 2021 were considered. All publishing years were included without any geographical restrictions. Articles that reported data not relevant to the study, reviews, and not written in English or French were excluded from the study.

### 2.5. Data Extraction

A data collection tool was designed in Microsoft Excel (Microsoft Corporation, USA) to capture data on different aspects of *E. abyssinica.* Three reviewers independently extracted relevant data from the included articles regarding the ethnobotany, ethnopharmacology, and phytochemistry of *E. abyssinica*. For ethnobotanical data, the diseases or ailments managed, parts used, and mode of preparation and administration were captured. For phytochemistry, the name of isolated pure compounds, chemical class, extraction solvent, and their efficacy and toxicity were captured. For ethnopharmacology, extraction solvent used, bioassay/model used, results of efficacy, and toxicity of extracts were captured. The collected data were checked for completeness and processed independently by two reviewers.

### 2.6. Data Analysis and Synthesis

Descriptive statistical methods were used to analyse the collected data. Results were expressed as percentages and frequencies and subsequently presented as tables and charts. The analyses were performed using SPSS statistical software (version 20, IBM Inc.).

## 3. Results and Discussion

### 3.1. Literature Search and Publications

**Figure 2:**
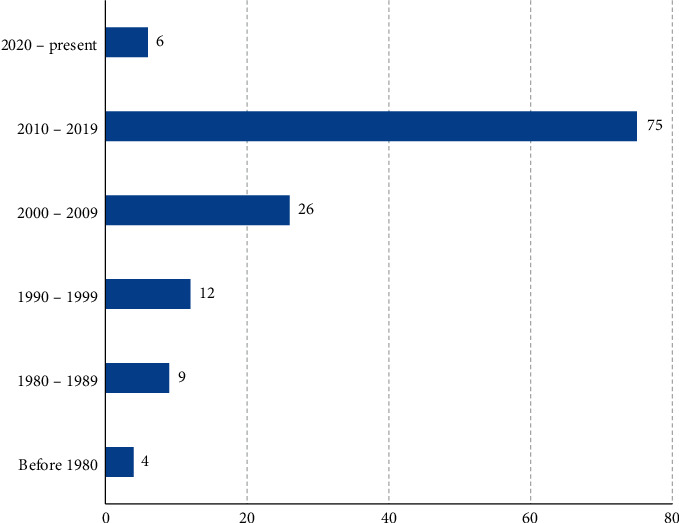
Number of reports on ethnomedicinal and nonmedicinal traditional uses, phytochemistry, pharmacology, and toxicity of *E. abyssinica* published up to date.

A total of 201 reports were retrieved out of which 132 met the inclusion criteria and were reviewed. Of these, 78 articles reported only on the ethnobotany, 27 articles on pharmacology only, 15 articles on both pharmacology and phytochemistry, 5 articles on phytochemistry only, and 3 articles on both ethnobotany and pharmacology while 4 articles on ethnobotany, pharmacology, and phytochemistry. Most of the articles (56.8%) were published in the 2010–2019 decade, indicating a lot of research is being done as compared to the preceding decades (Figure 2). This could be due to the (1) growing need for more effective and less toxic medicinal products of plant origin, (2) emerging antimicrobial resistance that has rendered most chemotherapeutic agents less effective, (3) new disease outbreaks like Ebola, and (4) increase in noncommunicable diseases such as cancers, hypertension, diabetes mellitus, and sexual dysfunction that require readily available, affordable, effective, and safe therapies.

### 3.2. Taxonomy, Morphology, Distribution, and Propagation

*Erythrina abyssinica* belongs to the kingdom Plantae, phylum Spermatophyta, subphylum Magnoliophyta (flowering plants), class Magnoliopsida (dicotyledons), order Fabales, family Fabaceae (legumes), subfamily Papilionoideae, genus *Erythrina* (L.), and species *abyssinica* (Lam ex. DC.). The frequently encountered synonyms of this species include *E. kassneri* Baker f., *Corallodendron suberifera* (Welw. ex Baker) Kuntze, *E. bequaerti* De Wild., *E. tomentosa* R. Br., *Chirocalyx abyssinicus* (Lam.) Hochst., and *C. tomentosus* Hochst. [3].

**Figure 3:**
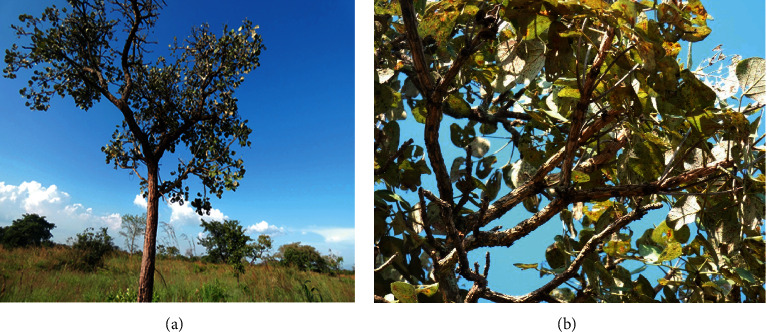
*Erythrina abyssinica*: (a) tree growing in its natural habitat and (b) leaves (photos taken by Samuel Baker Obakiro from Katakwi District, Eastern Uganda).

*Erythrina abyssinica* grows as a multibranched deciduous tree or shrub up to a height of 12–15 m tall usually with a rounded spreading crown (Figure 3). The branches have a corky thick deeply fissured bark with prickles (4–8 mm long). The leaves are trifoliate alternately arranged with long (6–20 cm) petiole. The leaflets can be ovate, cordate, and almost circular, rounded at the base and obtuse or notched at the apex, with network venation, dense hair usually at the abaxial surface, and prickles [49, 50]. The inflorescence is raceme, dense, pyramidal, and either terminal or axial with a long peduncle (up to 20 cm) and caducous bracts. Flowers are bisexual and papilionaceous having densely hairy, cylindrical, split at one side calyx, brightly coloured (orange to red) corolla with free keel petals, 10 fused and one free stamen, one carpel with a superior cylindrical oblong ovary, long style, and flat stigma head [51]. The fruits are linear-oblong pods, brown to black in colour, usually hairy, dehisce at two values to release ellipsoid, long (6–12 mm), and bright red seeds [52]. The tree is anchored firmly in the ground by a deep root system [13, 20].

**Figure 4:**
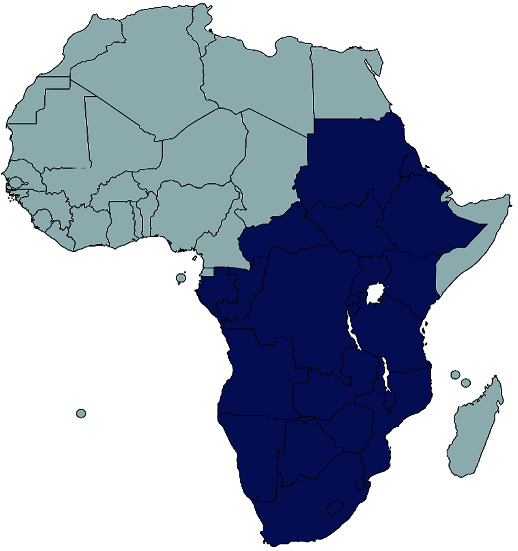
Native geographical distribution of *E. abyssinica* (based on retrieved literature [4, 10, 11, 15, 21, 23–25, 27–29, 58–63]).

*Erythrina abyssinica* can be propagated either using seeds, wildings [40], or cuttings, but the former has comparatively lower germination rates of 10–30% with propagation restricted to rainy seasons [3, 11, 53]. It grows naturally in woodland and wooded grasslands (savannah woodlands, grasslands, and scrublands, secondary scrub vegetation, regions with 500–2000 mm annual rainfall and optimal temperatures of 15–25°C) [11, 54–57]. Thus, it is widespread from Sudan, South Sudan, Uganda, Kenya, Rwanda, Burundi, Democratic Republic of Congo, Congo (Brazzaville), Tanzania to Ethiopia, Eritrea, Angola, Namibia, Botswana, Central African Republic, Swaziland, Lesotho, Gabon, Zambia, Zimbabwe, and Mozambique (Figure 4) [3, 10, 11, 53]. It has also been introduced as an ornamental in Mauritius and various places in Tropical Asia and Central America, including Afghanistan, Bangladesh, Bhutan, India, Nepal, Pakistan, and Sri Lanka [10, 53]. In South Sudan for instance, the tree grows at up to 2000 m altitude while in Tanzania, they are found at up to 2300 m. The tree naturally grows on loamy to clay soils, with preference for deep well-drained soils on plateaus and slopes with a pH of 3.5–5.5. The tree is termite- and fire-resistant primarily due to its deep root system but cannot tolerate frost, explaining its limited distribution in cold regions [11, 53].

### 3.3. Ecological, Traditional, and Medicinal Uses

*Erythrina abyssinica* being a legume is well known for fixing nitrogen into the soil and thus enhances soil fertility. Because of this, it plays an important role in phytorestoration and forest regeneration in polluted soils [64–66]. Its flowers also secrete nectar that is fed on by pollinating insects especially bees hence being important in both horticulture and apiculture [67]. Although this plant usually grows naturally in the wild, some communities cultivate it in their homesteads as an ornamental plant, for live fencing purposes due to its brightly coloured flowers and prickles, a material for dye, and craft materials such as curios and necklaces (from seeds) [9, 20, 68, 69]. The stem of this plant is also harvested to obtain timber and charcoal for furniture and energy purposes, respectively [20]. In livestock farming, the plant leaves are used as fodder for animals [5, 70, 71].

The stem bark, seeds, roots, root bark, leaves, and flowers of *E. abyssinica* and the whole plant either in combination or singly are used to prepare herbal remedies for various human ailments (Table 2). However, the stem bark and roots are the most commonly used parts in the preparation of herbal remedies. Even in efficacy, toxicity, and phytochemical studies, the stem bark and roots were the most investigated. This could probably be due to high yield associated with them because of their high potential in concentrating and storing phytochemicals. The seeds were indicated to be poisonous when crushed [11]. The commonest methods of preparation and administration of herbal medicines from this plant are boiling (decoctions) and then drinking, cold infusions (taken orally), pounding dried samples into powder and then licking, pounding fresh samples into a paste and applying topically, squeezing fresh samples and mixing with bathing water, or direct chewing of the different parts (Table 2).

Among the frequently reported ailments for which herbal medicines containing *E. abyssinica* are used include bacterial and fungal infections, malaria, leprosy, tuberculosis (cough), inflammatory diseases, HIV/AIDS, cancer, and metabolic disorders such as diabetes mellitus, obesity, and anaemia. Other conditions treated using this plant include snake bites, antagonizing poisons, venereal diseases (sexually transmitted diseases, e.g., gonorrhea, syphilis, and urinary tract infections including schistosomiasis), soft tissue and skin infections, diarrhea, infertility and pregnancy-related conditions, pneumonia, epilepsy, central nervous system- (CNS-) related disorders, vomiting, hepatitis, and helminthiasis. In ethnoveterinary medicine, extracts of *E. abyssinica* are used in the management of poultry and livestock diseases such as new castle disease, anaplasmosis, and helminthosis [43, 89, 119, 123, 124].

### 3.4. Phytochemical Profile of *E. abyssinica*

#### 3.4.1. Preliminary Phytochemical Analyses

Qualitative phytochemical screening of medicinal plants is an essential step to their detailed phytochemical and pharmacological investigation [125]. Preliminary phytochemical screening of different solvent extracts of *E. abyssinica* indicated the presence of tannins, saponins, alkaloids, and flavonoids as the main therapeutic secondary metabolites (Table 3).

#### 3.4.2. Structural Elucidation

Like in many natural product research studies, chromatography has been used in the isolation of compounds from crude extracts of *E. abyssinica.* The most widely used techniques included high-performance liquid chromatography (HPLC), gas chromatography (GC), high-performance thin-layer chromatography (HPTLC), and ultraperformance liquid chromatography (UPLC) [129]. Spectroscopic techniques such as mass spectrometry (MS), ultraviolet (UV) spectrophotometry, one-dimensional nuclear magnetic resonance (1D-NMR) spectroscopy, and its complementary techniques (heteronuclear multiple bond correlation (HMBC) spectroscopy, heteronuclear multiple quantum coherence (HMQC) spectroscopy, nuclear overhauser effect spectroscopy (NOESY), and circular dichroism (CD) spectroscopy) have been used to elucidate chemical structures of the isolated compounds [130]. Chromatography-spectroscopy hyphenated techniques have become more commonly used in recent decades due to the increased efficiency, sensitivity, and detection limits [1]. These include LC-MS, GC-MS, UPLC-MS, HPTLC-UV, HPLC-photodiode array detection, LC-NMR-MS, GC-NMR-MS, and high-resolution electron spray ionization (ESI)-MS [130].

**Figure 5:**
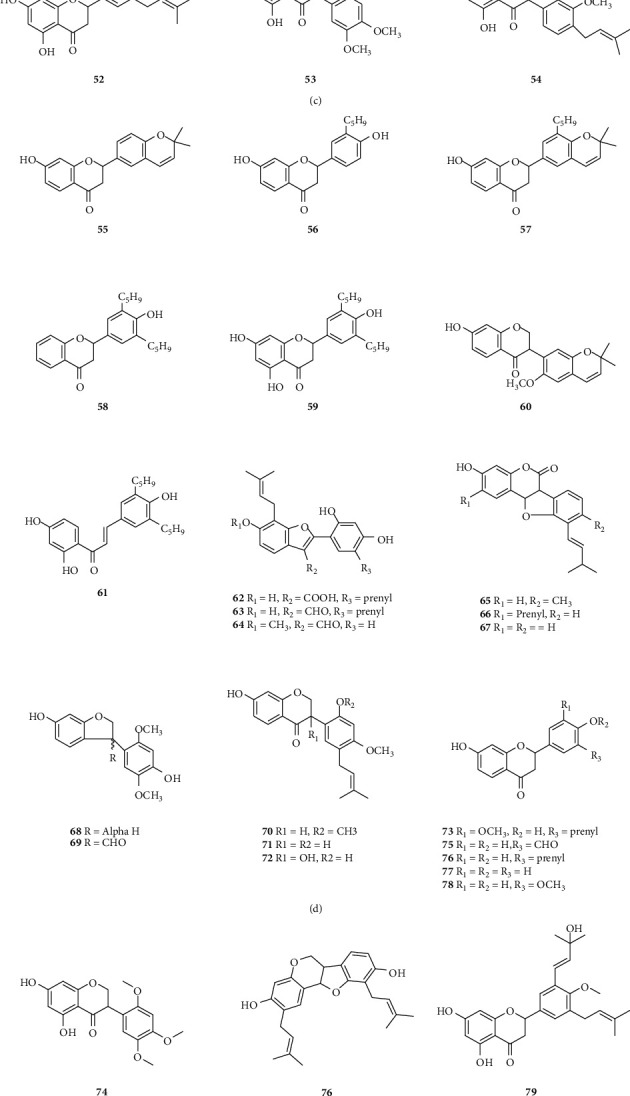
Chemical structures of the phytochemicals isolated from *E. abyssinica*. The numbers: **1–122** correspond to compounds mentioned in Table 4.

A total of 122 phytochemicals which are primarily alkaloids, flavonoids, and triterpenoids have been isolated from *E. abyssinica* (Figure 5; Table 4). Some of the isolated compounds are specific to *E. abyssinica* while others have been reported to be present in other species of the genus *Erythrina* [149]. Because genus *Erythrina* belongs to the family Fabaceae, its members have a rich diversity of secondary metabolites (phytochemicals) amongst themselves due to possession of various biosynthetic pathways [150]. However, some species share common phytochemicals, and hence, these act as biomarkers for nutraceutical, pharmacological, and toxicological potentials in the food and drug industries [130, 151].

*(1) Alkaloids*. In the present study, we retrieved thirteen alkaloids (**1–12** and **95**) that have been isolated from *E. abyssinica* (Table 4, Figure 5). The *Erythrina* alkaloids have a tetracyclic carbon skeleton with three rings (A, B, and C) common to all the alkaloids and the fourth ring (D) which varies among the different alkaloids [1, 152]. Lactonic alkaloids contain ring D as an unsaturated *δ*-lactone, dienoid alkaloids possess a benzenoid ring D (with two double bonds at C-1 and C-2, and C-6 and C-7), and alkenoid alkaloid possess a benzenoid ring D with a double bond between C-1 and C-6. Aromatic alkaloids and those containing a double bond at C-16 undergo stereoisomerism to give rise to other alkaloid derivatives [152].

*(2) Flavonoids*. A total of 106 flavonoids have been isolated and identified from *E. abyssinica*. These include five benzofurans, six chalcones, two coumestans, six isoflavones and seventy-two flavanones, four flavones, and eleven pterocarpans.

- *Benzofurans*. Benzofurans are heterocyclic compounds consisting of benzene and furan rings fused together. Five benzofurans (**65–69**) have been isolated from the stem bark of *E. abyssinica* [144].
- *Chalcones*. Chalcones, also known as chalconoids or benzyl acetophenones, are *α*, *β*-unsaturated ketones made up of two aromatic rings (designated as rings A and B) with diverse substituents. They possess conjugated double bonds and a completely delocalized *π*-electron system on both benzene rings. Chalcones have been widely known in medicinal chemistry as potential templates for the synthesis of therapeutic agents [153]. In this study, seven chalcones **(15**, **28–32, and 47**) were retrieved to have been isolated from the roots and stem bark of *E. abyssinica*.
- *Coumestans*. Coumestans are oxidized derivatives of pterocarpans consisting of a benzoxole fused to a chromen-2-one to form 1-benzoxolo[3,2-c]chromen-6-one. They are responsible for the phytoestrogenic activity of most medicinal plants of the family Fabaceae [154]. Two coumestans, erythribyssin N **(62)** and isosojagol **(64),** were isolated from the stem bark of *E. abyssinica.*
- *Isoflavones and Flavanones*. Isoflavones are a large group of flavonoids possessing a 3-phenylchroman skeleton that is biosynthetically obtained by rearrangement of the 2-phenylchroman flavonoid system. They are naturally occurring exclusively in the family Fabaceae (Leguminosae). Differences among isoflavones arise from the presence of extra heterocyclic rings, different oxidation states in this skeleton, and the number of substituents on the isoflavone moiety [155]. On the other hand, flavanones have the basic 2,3-dihydroflavone structure. They are distinguished from the rest of the flavonoid class by the lack of a double bond between C-2 and C-3 and the presence of a chiral center at C-2 position. Members differ from one another in the position and/or the number of the constituent methoxy and hydroxyl substituents [156]. Unlike isoflavones, flavanones are naturally occurring in members of family Fabaceae, Compositae, and Rutaceae. A total of six isoflavones **(25–27**, **83**, **110**, and **111)** and 72 flavanones **(14**, **17–22**, **24**, **33–46**, **48–61**, **63**, **70–75**, **77–82**, **84**, **87–92**, **100–103**, **108**, **109**, **118–119,** and **121–122)** have been isolated from *E. abyssinica* root bark, stem bark, and roots.
- *Pterocarpans*. Pterocarpans are structural analogs to isoflavonoids with a benzofurochromene skeleton. They can also be derived from coumestans through reduction reactions. They have two asymmetric centers at C-6a and C-11a and may exist as *cis* or *trans* isomers. The presence of pterocarpans has been attributed to their synthesis by members of the family Fabaceae in response to infections by microorganisms as defense molecules [157]. Eleven pterocarpans (**13**, **16**, **23**, **76**, **85**, **86**, **93**, **94**, and **112–114**) were isolated from the roots and root bark of *E. abyssinica* [133, 134, 136, 141].

*(3) Terpenoids (Sesquiterpenes and Triterpenoids)*. Sesquiterpenes are terpenoids with fifteen carbons (C15) consisting of three isoprene units. They are the dominant constituents of essential oils and other pharmacologically active oxygenated hydrocarbons occurring in higher plants. They naturally exist as hydrocarbons or oxygenated derivatives of hydrocarbons such as carbonyl compounds, alcohols, lactones, and carboxylic acids [158]. Three sesquiterpenes, 3,6-caryolanediol (**115**) and clovane-2,9-diol (**116**) along with caryolane-1,9-diol (**96**), were isolated from *E. abyssinica* roots [134]. On the other hand, two new triterpenoids, abyssaponin A (**97**) and abyssaponin B (**97**) along with a triterpenoid saponin, soyasapogenol B **(99),** were isolated from *E. abyssinica* stem bark [147].

### 3.5. Pharmacology of *E. abyssinica* and Isolated Compounds

In this section, we report investigations which evaluated the pharmacological potential of both extracts and isolated pure compounds from *E. abyssinica.* Indeed, phytochemicals in this species possess antibacterial, antifungal, antiviral, anticancer, antioxidant, anti-inflammatory, antimycobacterial, anti-HIV/AIDS, antiplasmodial, antihelmintic, antiobesity, antipyretic, antidiabetic, antianemic, and hepatoprotective bioactivities (Tables 4 and 5).

#### 3.5.1. Anti-Inflammatory Activity

The aqueous root bark of *E. abyssinica* at doses less than 100 mg/kg showed considerable *in vivo* anti-inflammatory activity against *Trypanosoma brucei*-induced inflammation in mice [50]. The extract-treated group had a lower number of astrocyte reactivity and reduced perivascular cuffing than the nontreated mice. It was suggested that the extracts reduced the infiltration of the inflammatory cells into the cerebrum of the brain. The anti-inflammatory activity was attributed to the alkaloids and flavonoids present in the extracts although the pure compounds responsible were not identified [50]. Interestingly, other crude extracts and pure compounds isolated from members of the genus *Erythrina* have been validated to possess good anti-inflammatory activities via different mechanisms. For example, the ethyl acetate and ethanol extracts of *E. latissimi, E. caffra*, and *E. lysistemon* showed good anti-inflammatory activity through reduction in the synthesis of prostaglandins as a result of inhibition of cyclooxygenase activity [168]. Erycristagallin isolated from *E. mildbraedii* inhibited leukotriene synthesis via the 5-lipoxygenase pathway, thereby demonstrating *in vitro* anti-inflammatory activity (IC_50_ = 23.4 *μ*M) in polymorphonuclear leukocytes [169]. Three flavonoids (abyssinone V, erycrystagallin, and 4′-hydroxy-6,3′,5′-triprenylisoflavonone) isolated from the methanolic stem bark extract *E. variegate* had strong phospholipase A2 (PLA2) inhibitory activity with IC_50_ values of 6, 3, and 10 *μ*M, respectively [170]. This implied that these compounds can significantly reduce the synthesis of arachidonic acid and consequently diminish the synthesis of prostaglandins and leukotrienes. Two prenylated flavanones (sigmoidin A and sigmoidin B) isolated from *E. sigmoidea* were reported to selectively inhibit 5-lipoxygenase but had no effect on cyclooxygenase-1 activity. Sigmoidin A had a dose-response inhibitory potency (IC_50_ = 31 mM). In the PLA2-induced mouse paw oedema assay, only the sigmoidin B inhibited oedema formation with a percentage inhibition of 59% compared to cyproheptadine (positive control) which had 74% after 60 minutes. In the TPA test, both compounds reduced the induced oedema by 89% and 83%, respectively. It was suggested that the compounds had different mechanisms of action depending on whether one or two prenyl groups were present in ring B of the flavonoid [83]. Since these same compounds have been isolated from *E. abyssinica*, it is highly probable that the reported anti-inflammatory activity of this plant is due to one or a combination of these mechanisms.

#### 3.5.2. Antioxidant Activity

The *in vitro* 2, 2-diphenyl-1-picrylhydrazyl (DPPH) radical scavenging assay has been widely used to evaluate the antioxidant activity of various phytochemicals and extracts. The ethanolic extract of *E. abyssinica* (10–320 *μ*g/mL) showed dose-dependent DPPH radical scavenging that was comparable to that of ascorbic acid (a known antioxidant) [159]. Abyssinone VII, sigmoidin B, eryvarin L, and 3-methylbutein isolated from the stem bark and root wood of *E. abyssinica* showed considerable DPPH radical scavenging potency (IC_50_ = 12–52 *μ*g/mL) although the standard antioxidants (ascorbic acid, gallic acid, and quercetin) had superior activity (IC_50_ = 4–18 *μ*g/mL) [134]. The acetone crude extract of the root bark of *E. abyssinica* (IC_50_ = 7.7 *μ*g/mL) and two isolated pterocarpenes, erycristagallin (IC_50_ = 8.2 *μ*g/mL) and 3-hydroxy-9-methoxy-10-(3,3-dimethylallyl) pterocarpene (IC_50_ = 10.8 *μ*g/mL), showed DPPH radical scavenging activity in a dose-dependent manner similar to that of quercetin (IC_50_ = 5.4 *μ*g/mL) [133]. The radical scavenging activity of these compounds is due to their free phenolic groups which can donate electrons to the radicals [171]. For flavonoids, the O-dihydroxyl structure in the B ring, the 2,3-double bond in conjunction with the 4-oxo function in the C ring, and the 3- and 5-hydroxyl groups with hydrogen bonding to the keto group are responsible for the antioxidant activity. In pterocarpans, the 3,3-dimethylallyl groups enhance the radical scavenging activities and also increase the lipophilicity of these compounds making them better antioxidants than polar flavonoids [133].

#### 3.5.3. Anticancer Activity

The chloroform, methanol, and ethyl acetate extracts showed cytotoxic activity against different tumor cells (cervical, liver, laryngeal, colon, and breast) and strongly inhibited protein tyrosine phosphatase (PTP1B) activity with IC_50_ ranging between 1 and 100 *μ*g/mL. Using the dimethylthiazol-2,5-diphenyl-tetrazolium bromide (MTT) assay, the abyssinones A–D and abyssaponins (A and B) isolated from *E. abyssinica* stem bark exhibited considerable cytotoxicity against MCF-7 and MDA-MB-231 breast adenocarcinoma cell lines with IC_50_ ranging between 12.9 and 74 *μ*M as compared to resveratrol (IC_50_ = 13.9–19.3 *μ*M) [147]. The mechanisms by which these phytochemicals mediated their anticancer activity were however not elucidated. However, related phytochemicals isolated from *E. suberosa* showed to induce apoptosis through the inhibition of NF-kB factor and via an increase in cytosolic cytochrome C that stimulates caspases 9 and 3 which further activates intrinsic apoptosis pathway [172].

#### 3.5.4. Antidiabetic and Antiobesity Activity

The aqueous extract of this plant showed significant antihyperglycemic activity at a dose of 500 mg/kg in rats using the oral glucose tolerance test (OGTT) with a hyperglycemia inhibition factor of 38.5% as compared to glibenclamide (49.6%). It was suggested that probably the inhibition of the SLGT-1 and GLUT-2 transporters along with *α*-glucosidase were the possible mechanisms for the antidiabetic activity [114]. In an acute OGTT, the ethanolic extract of *E. abyssinica* significantly decreased blood glucose levels in both normal and streptozotocin- (STZ-) induced diabetic rats in a dose-dependent manner (100, 200, and 400 mg/kg) when compared with negative (normal saline) and positive control (glibenclamide) [159]. In a subchronic antidiabetic test, daily oral administration of the same doses of extract for six weeks significantly lowered blood glucose levels in STZ-induced diabetic rats in a dose-dependent manner when compared with the diabetic control group. In this study, glibenclamide (5 mg/kg) significantly lowered blood glucose in nondiabetic rats only but not in diabetic rats [159].

Benzofurans, coumestans, and flavanones isolated from the stem bark of *E. abyssinica* had marked stimulation of the AMP-activated protein kinase (AMPK) activity with varying potencies at 10 *μ*M concentrations with coumestans and benzofurans showing the highest potency. The prenyl groups in coumestans and benzofurans were suggested to be responsible for the enhanced stimulatory activity while their substitution with a methoxy group in the B ring could be responsible for the decreased activation of the AMPK. Activated AMPK plays a critical role in glucose and lipid metabolism such as enhancing insulin sensitivity, stimulating glucose uptake in the muscles, suppressing gluconeogenesis in the liver, increased oxidation of fatty acids oxidation, and diminished fatty acid synthesis. All these mechanisms are responsible for the antidiabetic activity of the isolated phytochemicals [144]. Further, prenylated flavanones from the stem bark of *E. abyssinica* inhibited protein tyrosine phosphate 1B (PTP1B) activity in an *in vitro* assay with IC_50_ values ranging from 15.2 to 19.6 *μ*M compared to RK-682 (positive control, IC_50_ = 4.7 *μ*M). Since PTP1B regulates the insulin and leptin signaling pathways, its inhibition has been reported to result in hypoglycemic effect, and hence, its inhibitory compounds have a great potential in acting as antidiabetic and antiobesity agents [135, 142, 160]. Sigmoidin A, a flavanone isolated from the stem bark of *E. abyssinica* showed a considerable *in vitro* inhibitory activity on pancreatic lipase (IC_50_ = 4.5 *μ*M) and *α*-glucosidase enzyme (IC_50_ = 62.5 *μ*M). Although orlistat (an antiobesity drug) exhibited a superior inhibitory activity against pancreatic lipase (IC_50_ = 0.3 *μ*M), the observed activity suggested that prenylated flavonoids have potential antilipase activity and hence could be antiobesity agents. Interestingly, its *α*-glucosidase inhibitory potency was better than that of acarbose (IC_50_ = 190.6 *μ*M), a standard antidiabetic agent [146].

#### 3.5.5. Antiparasitic Activity

The antiplasmodial activity of *E. abyssinica* has been evaluated using the nonradioactive antiplasmodial (*in vitro*) and four-day *Plasmodium berghei* ANKA suppressive (*in vivo*) bioassays [163]. The ethyl acetate extracts had strong *in vitro* antiplasmodial activity against chloroquine-resistant and chloroquine-sensitive *Plasmodium* strains with IC_50_ values of 5.3 and 7.9 *μ*g/mL, respectively [49, 163]. Subsequently, isolated chalcones, flavanones, and isoflavonoids had promising antiplasmodial activity against chloroquine-sensitive and chloroquine-resistant *P. falciparum* strains with IC_50_ ranging from 4.9 to 24.9 *μ*M although chloroquine still had superior activity [49].

Another earlier *in vitro* study by Kebenei et al. [143] assessed the possible use of artemisinin in combination with a potential antimalarial drug from ethyl acetate extract of *E. abyssinica* stem bark reported that abyssinone V isolated from the extract was effective against chloroquine-sensitive (D6) *P. falciparum* parasites with IC_50_ value of 3.19 *μ*g/mL. The interaction of artemisinin and abyssinone V analyzed using combination ratios of 10 : 90 to 90 : 10, respectively, against *P. falciparum* led to the identification of an antimalarial combination therapy of artemisinin and abyssinone V with sum of fraction inhibiting concentration (FIC) of 0.79 at a ratio of 2 : 3, respectively [143].

In an *in vivo* study, the root extracts of this plant suppressed *P. berghei* infection by 77%, 71%, and 48% in mice treated at 50, 25, and 12.5 mg/kg, respectively. It was also found out that the mice treated with a higher dose (50 mg/kg) had a significantly longer survival time than those treated with lower doses and even chloroquine [164]. The crude leaf extracts of *E. abyssinica* had weak activity against *P. falciparum* chloroquine-sensitive Sierra Leone I (D6) and multidrug-resistant Indochicha I (W2) strains with IC_50_ ranging from 165 to 468 *μ*g/mL [145]. Conversely, erythinasinate A and 7-hydroxy-4′-methoxy-3-prenylisoflavone isolated from *E. abyssinica* methanolic leaf extract had moderate antiplasmodial activity against W2 and D6 with IC_50_ between 120 and 150 *μ*g/mL [145]. Isolated compounds had a much higher antiplasmodial activity than the crude extract. Isolation removes matrix interference and increases the concentration of the active ingredient at the drug target [173]. In another study, the ethyl acetate extract of this plant at 10 *μ*g/mL inhibited the growth of *P. falciparum* by 83.6% as compared to chloroquine (98.1%) [73]. This antiplasmodial activity was also confirmed in *E. burttii*, a related species. The acetone root bark extract of *E. burttii* had good *in vitro* antiplasmodial activity against the chloroquine-resistant and chloroquine-sensitive *P. falciparum* strains with IC_50_ of 1.73 and 0.97 *μ*g/mL, respectively [163]. The methanolic leaf extract of *E. abyssinica* also exhibited moderate mosquitocidal and larvicidal activities with 65.5% and 65.1% mortality and corresponding IC_50_ values of 231.90 and 218.90 mg/mL, respectively. However, the activities were lower compared to that of the standard drug temephos (99.90 %) [49, 145].

The antihelmintic activity of *E. abyssinica* has been validated using the worm motility assessment assay on *Ascaridia galli.* The ethanolic leaf extract of this plant at increasing doses up to 50 mg/mL had good antihelmintic activity against *A. galli* comparable to that of piperazine [124]. At 50 mg/mL, the extract immobilized 95% of the worms as compared to 100% of the standard drug. In another study, 5% concentration of the extract killed all the worms after 48 hours [120]. Although the active phytochemicals were not identified, it was suggested that the antihelmintic activity of this plant could be due to tannins and alkaloids present in the crude extracts. This is because tannins are polyphenolic compounds like some synthetic antihelmintic drugs such as oxyclozanide and niclosamide. Therefore, the tannins could in a similar way interfere with energy release in the worms through uncoupled oxidative phosphorylation. But also, the tannins could bind to free proteins in the gastrointestinal tract or glycoprotein on the cuticle of the helminth, thereby impairing food absorption, motility, and reproduction. On the other hand, alkaloids being able to stimulate excitatory cells could cause neurological dysfunction that result in paralysis and consequent death of the parasites [124].

#### 3.5.6. Antibacterial and Antifungal Activities

The antibacterial and antifungal activities of the crude extracts and isolated compounds of *E. abyssinica* have been widely evaluated using the microbroth dilution assay against various pathogens. The bacteria tested against included *Escherichia coli, Staphylococcus aureus, Bacillus subtilis,* methicillin-resistant *Staphylococcus aureus, Pseudomonas aeruginosa, Klebsiella pneumoniae, Salmonella typhi,* and *Bacillus cereus* while the fungi were *Micrococcus luteus, Candida utilis, Candida albicans, Mucor mucedo, Saccharomyces cerevisiae, Penicillium crustosum, Microsporum gypseum, Trichophyton mentagrophytes,* and *Cryptococcus neoformans.* The hexane, dichloromethane, ethyl acetate, methanol, and ethanol extracts of this plant showed antibacterial and antifungal activities with minimum inhibitory concentrations (MICs) between 3 and 10,000 *μ*g/mL against different pathogens. Generally, the extracts had strong activity against Gram-positive bacteria and moderate to weak activity against Gram-negative bacteria [100, 123, 141, 145, 167, 174, 175]. It was suggested that this could be due to the unique cell wall of Gram-negative bacteria which consists of an additional lipopolysaccharide layer and periplasmic space that make it difficult for antibiotics to penetrate into them. The wide variation in the MIC values could be due to the difference in the resistance profiles of the tested microorganisms with those strains that are more resistant having higher values of MIC compared to the sensitive strains. Although standard drugs had superior activity, isolated pure compounds had higher activity (slightly lower MIC values) than the crude extracts. Flavonoids from the stem bark had MIC ranging between 0.3 and 10 *μ*g/mL against *B. subtilis, S. aureus, E. coli,* and *S. cerevisiae* as compared to the antibacterial chloramphenicol (MIC = 0.001–0.5 *μ*g/mL) and antifungal miconazole (MIC = 0.005 *μ*g/mL) [134]. Two pterocarpans and eight flavonoids isolated from the root bark had significant activity against *S. aureus* and *B. subtilis* with MIC ranging between 6.25 and 50 *μ*g/mL. But moderate activity against many Gram-negative bacterial and fungal strains with MIC greater than 100 *μ*g/mL [141]. Phaseolin and erythrabyssin I showed significant antifungal activity (MIC = 6–50 *μ*g/mL) against *S. cerevisiae*, *C. utilis, R. chinensis*, and *M. mucedo* [136]. In a recent study, Schultz et al. [176] reported that ethyl acetate and ethanolic extracts of *E. abyssinica* bark did inhibit *Enterococcus faecium* EU-44 (IC_50_ = 64 *μ*g/mL and MIC > 256 *μ*g/mL), *Staphylococcus aureus* UAMS-1 (IC_50_ = 32 *μ*g/mL and MIC 64 *μ*g/mL), *Acinetobacter baumannii* CDC-0033 (IC_50_ = > 256 *μ*g/mL and MIC > 256 *μ*g/mL) but had no activity against *Klebsiella pneumoniae* CDC-004, *Pseudomonas aeruginosa* AH-71, and *Enterobacter cloacae* CDC-0032. Further, the extracts did not exhibit quorum sensing above 40% at 16 *μ*g/mL in a quorum-sensing inhibition plant extract library screen on *S. aureus* accessory gene regulator I reporter strain [176]. No study reported the mechanism of action of either the extracts or isolated compounds. Therefore, it remains to be determined whether the phytochemicals are microbiostatic or microbicidal.

#### 3.5.7. Antimycobacterial Activity

The crude methanolic root extract of *E. abyssinica* showed considerable antimycobacterial activity on the rifampicin-resistant (TMC-331) and pan-sensitive (H37Rv) *Mycobacterium tuberculosis* strain with a MIC of 2.35 mg/mL and 0.39 mg/mL, respectively. The MICs for isoniazid were 9.38 and 0.25 *μ*g/mL for TMC-331 and H37Rv, respectively [126]. In another study using the automated BACTEC Mycobacterial Growth Indicator Tube (MGIT) 960 TB system, the methanolic root bark of this plant inhibited the growth of four Mycobacterial strains (*M. tuberculosis, M. smegmatis*, *M. kansasii*, and *M. fortuitum*) at a concentration of 2 mg/mL. Isoniazid, a standard antitubercular drug had a growth inhibitory concentration of 0.5 mg/mL [177]. In a synergistic interaction study, the methanol and ethanol extracts of *E. abyssinica* (0.49 *μ*g/mL) when combined with either rifampicin or isoniazid (0.01 *μ*g/mL) had a complete inhibitory effect on the growth of *M. tuberculosis* (H37Rv). The standard drugs and methanol and ethanol extracts at the same tested concentration had innumerous, 125 and 10 colony-forming units [166]. It was postulated that probably the flavonoids, alkaloids, tannins, and terpenoids present in the extracts interacted with the standard drugs at the drug target levels, hence enhancing the activity of each other. The confirmed synergism could be used to explain the concomitant use of herbal medicines alongside the conventional therapies but also reaffirms the benefit of combination therapy in the management of susceptible and resistant tuberculosis. Despite the widespread use of *E. abyssinica* in the traditional management of tuberculosis, we did not find any reports on isolation and characterization of compounds from this plant against *M. tuberculosis*.

#### 3.5.8. Antiviral Activity

The anti-HIV-1 activity of this plant was evaluated using the MTT method. The alkaloidal fraction showed cytotoxicity of HIV-1-infected MT-4 cells with an IC_50_ of 53 *μ*M compared to efavirenz which had an IC_50_ of 45 *μ*M. The anti-HIV activity was attributed to the isoquinoline-type alkaloids present in the fraction that inhibit the HIV-1 replication through inhibition of viral entry and reverse transcription processes [59]. The other antiviral activities of this plant have not been validated. However, erysodine, erysotrine, and erythraline isolated from *E. crista-galli* but also present in *E. abyssinica* showed significant antiviral activity against tobacco mosaic virus (TMV) with IC_50_ of 1.48, 1.28, and 1.52 *μ*M, respectively, using the leaf disc method. The positive control ningnanmycin had an IC_50_ of 0.18 *μ*M [178]. Of great interest was the new alkaloid glycoside, erythraline-11-*β*-O-glucopyranoside which showed a much superior antiviral activity (IC_50_ = 0.59 *μ*M) against TMV as compared with its aglycone, erythraline (IC_50_ = 1.52 *μ*M).

#### 3.5.9. Antianaemic and Hepatoprotective Activity

The haematinic activity of this plant was evaluated in mice using the phenyl hydrazine-induced anaemic mice model. At doses less than 100 mg/kg, the aqueous stem bark extract of *E. abyssinica* significantly increased the diminished levels of haemoglobin (Hb), red blood cells (RBCs), and packed cell volume (PCV) in mice at the end of four weeks following daily oral administration of the extract. On the other hand, the extract did not have a significant effect on the levels of white blood cells, mean corpuscular volume, mean corpuscular haemoglobin, and other differentials. The observed antianaemic activity was attributed to the flavonoids, alkaloids, and cardiac glycosides present in the aqueous extracts. However, isolation and characterization were not done to identify the pure compounds responsible for this activity.

The hepatoprotective effect of the extract was evaluated using the nonalcoholic fatty liver disease (NAFLD) model on rats fed on high-fat and glucose diet. The water extracts at daily oral doses of 200 and 400 mg/kg for 56 days showed significant inhibitory effects against the development of nonalcoholic fatty liver disease. The extract was hepatoprotective against steatosis, inflammation, and hepatic ballooning. The extracts also significantly altered other hepatic-related biochemical indices as compared to standard drug pioglitazone [162]. This hepatoprotective activity was attributed to the coumestans, benzofurans, and pterocarpans present in the water extracts that regulate the activity of AMP kinases and protein tyrosine phosphatase 1B.

#### 3.5.10. Antipyretic and Estrogenic Activity

The estrogenic activity of this plant was studied using the smart button data loggers' model in ovariectomized rats. The methanol extract (200 mg/kg) and estrogen (1 mg/kg) reduced the number and frequency of hot flushes (171) as compared to those ovariectomized rats that did not receive the extract (264). Also, the rats treated with extract and estrogen had significantly reduced durations (683 and 869 minutes, respectively) of hot flashes than the untreated rats (1935 minutes). Thus, the methanol extract seemed to offer protection against small temperature rises which trigger hot flashes in the ovariectomized untreated rats. Although the real chemicals in the extract responsible for the antipyretic activity were not identified, it was postulated that the chemicals mimic estrogen by increasing the sweating threshold and thermoneutral zone size [161]. In a related study, the estrogenic activity of the erythroidines isolated from *E. poeppigiana* was evaluated using various estrogen receptor- (ER-) dependent test systems. These included the receptor binding affinity and cell culture-based ER-dependent reporter gene assays. It was found out that both *α*-erythroidine and *β*-erythroidine showed significant binding affinity values for ER*α* of 0.015 % and 0.005%, respectively, whereas only *β*-erythroidine bound to ER*β* (0.006 %). In reporter gene assays, both erythroidines showed a significant estrogenic stimulation of ER-dependent reporter gene activity in osteosarcoma cells that was detectable at 10 nM in a dose-dependent manner [179]. These erythroidines have also been reported to be present in *E. abyssinica* and thus could be responsible for the estrogenic activity of this plant.

#### 3.5.11. Anticonvulsant and Anxiolytic Activity

The long-known neuropharmacological activity of this plant was the curariform activity which is largely attributed to alkaloids present in it. Erysodine and erysopine isolated from the seeds of *E. abyssinica* showed significant curare-like activity both *in vitro* and *in vivo* [132]. The other CNS demonstrated activities of compounds present in *E. abyssinica* include anticonvulsant [180, 181], analgesic [180], and anxiolytic. In another study, erysodine and erysothrine (0, 3, or 10 mg/kg) administered orally exhibited anxiolytic effect in mice with comparable efficacy to diazepam (2 mg/kg) administered intraperitoneally. Using the elevated plus maze (EPM) model, only erysodine (10 mg/kg) increased the percentage of open arm entries and open arm time. In the light-dark transition model (LDTM), both erysothrine and erysodine demonstrated anxiolytic-like activity. However, while erysodine (10 mg/kg) increased both times spent in the illuminated compartment and the number of transitions between compartments, erysothrine (3 mg/kg) increased the number of transitions only. It was further observed that none of the two alkaloids neither altered the locomotory behaviour (i.e., the number of closed arm entries) of the animals in the EPM [182].

### 3.6. Toxicity Profile of *E. abyssinica*

Toxicological evaluation of medicinal plants, isolated pure compounds, and corresponding herbal products is one of the key requirements for their approval and licensing as pharmaceutical products by regulatory authorities. This is because apart from possessing pharmacological activity that can be exploited for therapeutic benefits, the same phytochemicals may interact with the same or different receptors and elicit toxicity. Some toxicities may either be dose-dependent or dose-independent. On the other hand, some may be immediate while others delayed. Although no substance can be declared to be completely devoid of toxicity, toxicity tests (acute, subacute, subchronic, and chronic) are used to determine the relative toxicity of potential therapeutic agents.

Despite the huge data regarding the pharmacological potential of *E. abyssinica*, there is a paucity of data regarding its toxicity. The seeds are traditionally known to be poisonous [11]. In an *in vitro* acute toxicity assay using the brine shrimp lethality model, the methanolic and ethanolic extracts of *E. abyssinica* had LC_50_ ≥ 1000 *μ*g/mL [127] and 997 *μ*g/mL [159], respectively. A related *in vitro* study using the haemolytic assay reported that the hexane (62.5 *μ*g/mL), dichloromethane (62.5 *μ*g/mL), ethyl acetate (62.5 *μ*g/mL), and methanol (125 *μ*g/mL) extracts of this plant showed low percentage haemolysis (15.5, 9.1, 15.4, and 39.7%) of red blood cells [175]. The higher percentage haemolysis observed with the methanol extract was attributed to the higher concentration of methanol extract. These *in vitro* results indicated that the extracts were safe within 24 hours of administration.

In a study which determined the *in vivo* acute toxicity of crude extracts from this plant, it was found out that the median lethal dose (LD_50_) of leaf and stem bark extracts was above 300 mg/kg body weight. All the mice orally administered with the extracts (100, 200, and 300 mg/kg) survived up to 72 hours and there were no significant behavioural changes between the treatment and control groups [183]. In another study, the methanolic root extract was found to have an oral LD_50_ of 776.2 mg/kg in mice [126]. As with the previous study, acute toxicity signs became more apparent at the highest doses. But still they were limited to sedation and reduced motor activity. Based on the OECD 2001 guidelines, since the LD_50_ is greater than 300 mg/kg, it can be inferred that the crude extracts are weakly toxic within 24 hours of a single high dose [184]. It is important to know that the seeds of *E. abyssinica* contain curare-like alkaloids. Thus, it is believed that, at high doses, these may cause anaesthesia, paralysis, and even death by respiratory failure [185].

In a subacute toxicity evaluation of the extract from this plant, the mice were dosed with 100, 200, and 300 mg/kg of the extract daily for 30 days. There was no significant difference in behaviour and physical and general activity parameters such as water intake, food consumption, and body weight between the treated groups and control group (no extract given) throughout the period of the experiments [183]. However, there were variations in biochemical parameters between the *E. abyssinica*-treated groups and nontreated group although it was not statistically significant. Particularly the treated group had higher levels of urea and creatinine and lower levels of potassium and sodium. There was also high total and/or conjugated bilirubin associated with *E. abyssinica*-treated groups. This could probably suggest possible liver insufficiency or interference with bile flow. However, this finding was inconclusive as it could be due to other contributing factors other than the liver. Another study reported that the *E. abyssinica* (1000 mg/kg) significantly increased the levels of urea and creatinine and level of serum diagnostic enzymes particularly alkaline phosphatase, lactate dehydrogenase, gamma-glutamyltransferase, and alpha-amylase in treated mice after 28 days of daily oral administration [128]. This probably indicated some degree of impairment of renal, liver, and heart functions. Histopathological evaluation of the tissues of the liver revealed necrotic foci, dilated and congested blood vessels, numerous hepatocytes with double nuclei in view, and infiltration of inflammatory cell, while the kidney tissues showed necrotic foci in the papillary region, loss of tubules in necrotic foci, and vacuolated cells in place of original cells. The liver being the primary detoxifying organ of the body while the kidney being the excretory organ are highly susceptible to damage by phytochemicals present in the extracts/herbal medicines.

The haematological parameters were also slightly altered by extract administration, suggesting an effect on the hematopoietic tissue [183]. As with the biochemical parameters, the assays did not conclusively show haemolysis or other blood-related toxicity of the extracts. In contrast, another study found out that the stem extract (1000 mg/kg) did not significantly alter the haematological indices of the treated rats as compared to the nontreated after 28 days of daily oral administration [128]. It can therefore be inferred that extracts of this plant have minimal toxicity effect on the hematopoietic tissue. Since this plant has been reported to have minimal toxicity on the liver, kidney, and hematopoietic tissue, it should be used with caution in traditional medicine. More evidence regarding its chronic toxicity is needed to guarantee its safety especially in the management of chronic conditions.

### 3.7. Clinical Studies

We did not find any relevant report reporting results of a clinical trial on either a pharmaceutical product or an herbal product from *E. abyssinica.* This could be probably due to the huge financial requirement to conduct clinical trials but also other challenges surrounding herbal medicine use.

## 4. Conclusion and Future Perspectives

*E. abyssinica* has been proven to harbor useful pharmacologically active phytochemicals against various diseases with significant efficacies although with some minimal toxicity profiles. There is therefore a need to generate more toxicological data about this plant and different isolated phytochemicals so as to generate sufficient evidence as regards their safety for human use. Once proven safe, the plant could provide a cheap and sustainable source of novel molecules for the development of new therapeutic agents for human ailments. To the best of our knowledge, we did not find any *E. abyssinica*-based pharmaceutical products in the literature, different pharmacopoeia, and drug development pipeline. The active phytochemicals identified could therefore be prioritized and/or optimized for further drug development. There is also a need to standardize and promote rational herbal medicine use through encouraging registration and licensing of products with proven efficacy and safety. Clinical studies utilizing extracts and isolated compounds from *E. abyssinica* are required. Due to its ethnomedicinal purposes, communities should be sensitized and encouraged to conserve this plant species.

## Figures and Tables

**Table 1:** Local names of *Erythrina abyssinica* used across African communities.

Folk name (local language)	Country	Authors
*Ejjirikiti* (Luganda), *Murinzi*, *Kiko Omoko/Echuko* (Rutoro, Rukonzo), *Oluo* (Lugbara), *Kisoro, Lochoro, Oding, Loting* (Acholi), *Kikiri* (Kwamba), *Engosorot* (Ateso), *Olawu* (Madi), *Koli* (Jopadhola), *Owila kot* (Lango), *Muyirikiti*, *Ekilama* (Lusoga), *Cheroguru*, *Muragolo* (Lugishu), *Mutembetembe* (Lugwe), *Bwiko* (Lukiga), *Kaborte* (Sebei), *Kiko, Muko* (Lunyangkore, Lutoro), *Mudongodongo, Mukobe* (Lunyuli)	Uganda	[2, 3, 10–15]
*Omotembe* (Kisii), *Muhuti* (Kikuyu), *Ekirikiti* or *Ol*-*Goroshe* (Maasai), *Muuti* (Meru), *Kivuti* or *Muvuti* (Kamba), *Mulungu* (Taita), *Mwamba ngoma*, *Mbamba ngoma*, *Muhuti*, *Mjafari* or *Mwamba* (Kiswahili), *Kumurembei* (Luhya)	Kenya	[10, 16–19]
*Qanqari* (Iraqw), *Mriri* (Chagga), *Muhemi* (Hehe), and *Muungu* (Pare), *Kisebhe* (Rungwe)	Tanzania	[20–22]
*Kuara*, *Korra, Korch* (Amharic)	Ethiopia	[10]
*Umuko* (Lunyarwanda)	Rwanda	[23–26]
*Dus* (Arabic), *Hab al Arous*	Sudan, South Sudan	[10, 27, 28]
*Chisunga* (Lunda)	Democratic Republic of Congo	[10]
*Mulunku* (Chokwe)	Angola	[4]
*Mulunguti, Mwale* (Nyanja)	Mozambique, Zimbabwe, Zambia, Malawi	[10]
*Mulunguti* (Bemba, Tongan)	Zambia, Mozambique, Zimbabwe	[5, 10]
*Mutiti* (Shona)	Zimbabwe	[5]
*Suwawue, Soaueh* (Tigrigna)	Eritrea, Ethiopia	[10, 29]

**Table 2:** Ethnobotanical uses of *Erythrina abyssinica* reported in the literature.

No.	Disease/ailments treated	Parts used	Method of preparation and administration	Country	Authors
1	Malaria, fevers	R, SB, L, F	Boiled and taken orally	Uganda, Kenya, Tanzania, Ethiopia, Eritrea, DR Congo, Sudan, Rwanda	[9, 13, 18, 21, 24, 28, 58, 72–82]
2	Inflammatory disorders, eye problems, and pain	SB, R, Sd	Boiled and taken orally; powdered, mixed with petroleum jelly, and smeared on the wound/swollen part. For eye problems, it is applied as liniment	Uganda, Tanzania, Kenya, South Sudan	[13, 19, 20, 27, 72, 83–88]
3	Bacterial and fungal infections	SB, L, F, WP	Decoction taken orally; powdered and licked; sliced bark chewed; cold infusion taken orally	Uganda, Kenya, Burundi	[13, 72, 89–91]
4	Skin and soft tissue infections, leprosy, and wounds	SB, F, L	Boiled in petroleum jelly and smeared at the tissue, herbal bath of infected skin part	Uganda, Kenya, Zimbabwe, Rwanda	[20, 24, 72, 81, 87, 92–95]
5	Tuberculosis (cough)	SB, R, L, F	Decoction taken orally; powdered and licked	Uganda, Kenya, Tanzania, Burundi, Zimbabwe	[31, 61, 72, 73, 95–99]
6	Cancer	SB, L, F	Boiled and taken orally	Uganda, Kenya	[39, 72, 100]
7	HIV/AIDS	SB, R, L	Decoction taken orally	Uganda, Kenya, Tanzania	[2, 39, 72, 98, 101–103]
8	Infertility, birth control, pregnancy related conditions	SB, R	Decoction, squeezing, chewing, taken orally	Uganda, Kenya	[31, 72, 73, 104–106]
9	Blood disorders (anaemia and jaundice)	R, SB, L, F	Boiled and taken orally	Uganda, Kenya, Tanzania	[27, 31, 72, 84, 107–109]
10	Venereal diseases	SB, L, F, RB	Boiled and taken orally	Uganda, Kenya, Zimbabwe, Rwanda	[19, 20, 63, 72, 87, 92, 100, 105, 110–112]
11	Diabetes mellitus	SB, L	Boiled and taken orally	Uganda	[72, 113, 114]
12	Hepatitis, measles, scabies, herpes, mumps, liver diseases	SB, R, L	Decoction and cold infusions taken. Dried leaf ash is mixed with oil or butter and applied externally to treat scabies	Rwanda, Kenya, Uganda, Tanzania	[22, 23, 101, 115]
13	Pneumonia	SB	Boiled in water and taken orally	Kenya	[92, 100]
14	Convulsions and CNS disorders	SB	Decoction, pound, and add salt	Uganda	[31]
15	Gastrointestinal disorders (diarrhea, stomach ache, vomiting, constipation, ulcers, dysentery, colic)	SB, R, L	Boiled, honey added, and taken orally. Decoction taken, or pounded, salt added, and taken. Root decoction with *Rhamnus prinoides* roots taken for colic. Decoction of young roots taken for constipation in children	Uganda, Kenya, Tanzania, Eritrea, Angola, Rwanda	[4, 19, 26, 29, 31, 87, 92, 101, 106, 107, 116–118]
16	Helminthiasis	SB	Decoction taken orally	Uganda, Kenya, Tanzania	[87, 105, 119, 120]
17	Snake bites/antidote for poisoning	R, SB, RB	Sap used/pounded and applied at the bite. Boiled and taken orally	Uganda, Kenya, Tanzania	[15, 16, 19, 109, 121, 122]

Parts used: L: leaves, R: roots, RB: root bark, Sd: seeds, SB: stem bark, F: flowers, and WP: whole plant.

**Table 3:** Some secondary metabolites reported in *E. abyssinica extracts*.

Secondary metabolites	Parts used	Solvent used	Yield (%)	Authors
Tannins, saponins, alkaloids, and flavonoids	Bark	Hexane	2.0	[60]
Alkaloids, terpenoids, saponins, tannins, and flavones	Root bark	Methanol (crude)	Not reported	[126]
Alkaloids, saponins, cardiac glycosides, coumarins, and anthraquinone derivatives	Roots	Methanol	23.6	[127]
Alkaloids, flavonoids, tannins, and cardiac glycosides	Stem	Water	0.34 (alkaloidal and flavonoid content)	[128]
Alkaloids, flavonoids, terpenoids, and saponins	Stem bark	Methanol	4.82	[62]

**Table 4:** Phytochemical composition and pharmacological activities of compounds isolated from different parts of *Erythrina abyssinica*.

Name of the compound identified	Chemical class	Part used	Solvent used	Techniques used	Bioactivity tested	Result	Authors
(+)-Erysotrine **(1)**	Alkaloid	NS	NS	NMR	Not tested	Not applicable	[131]
(+)-Erythravine **(2)**	Alkaloid	NS	NS	NMR	Not tested	Not applicable	[131]
(+)-Erythristemine **(3)**	Alkaloid	NS	NS	NMR	Not tested	Not applicable	[131]
(+)-Erysovine **(4)**	Alkaloid	NS	NS	NMR	Not tested	Not applicable	[131]
(+)-Erysodine **(5)**	Alkaloid	Sd	Chloroform, ethanol	NMR	Curare-like activity	Strong activity	[131, 132]
(+)-Erysopine **(6)**	Alkaloid	Sd	Chloroform, ethanol	NMR	Curare-like activity	Strong activity	[131, 132]
(+)-Erythraline **(7)**	Alkaloid	NS	NS	NMR	Not tested	Not applicable	[131]
(+)-8-Oxoerythraline **(8)**	Alkaloid	NS	NS	NMR	Not tested	Not applicable	[131]
(+)-11-Oxoerysodine **(9)**	Alkaloid	NS	NS	NMR	Not tested	Not applicable	[131]
(+)-11-Methoxyerysovine **(10)**	Alkaloid	NS	NS	NMR	Not tested	Not applicable	[131]
(+)-Erythratidine **(11)**	Alkaloid	NS	NS	NMR	Not tested	Not applicable	[131]
(+)-Erythratine **(12)**	Alkaloid	NS	NS	NMR	Not tested	Not applicable	[131]
8-Methoxyneorautenol **(13)**	Pterocarpan	RB	Acetone	HRMS, NMR, HMBC	Radical scavenging properties	Moderately active	[133]
Eryvarin L **(14)**	Benzofuran	Rt	Chloroform: methanol (1 : 1)	UV, NMR, EI-MS, HMBC	Antimicrobial and antioxidant activities	Good antioxidant activity	[134]
Licoagrochalcone A (**15**)	Chalcone	Tw	Chloroform: methanol (1 : 1)	UV, NMR, EI-MS, HMBC	Antimicrobial and antioxidant activities	Good radical scavenging activity	[134]
3-Hydroxy-9-methoxy-10-(3,3-dimethylallyl) pterocarpene **(16)**	Pterocarpan	RB	Acetone	HRMS, NMR, HMBC	Radical scavenging properties	Very active	[133]
(2S)-5,7-Dihydroxy-3′-prenyl-2″*ξ*-(4″-hydroxyisopropyl) dihydrofurano[1″,3″ : 4′,5′] flavanone **(17)**	Flavanone	SB	Methanol	UV, HPLC, NMR, HMQC, HMBC	PTP 1B inhibitory activity	No activity	[135]
(2S)-5,7-Dihydroxy-3′-prenyl-2″*ξ*-(4″-hydroxy-isopropyl)-3″-hydroxy-dihydrofurano[1″,3″:4′,5′]flavanone, and (2S)-5,7,3′-trihydroxy-2′-prenyl-2″*ξ*-(4″-hydroxyisopropyl)-3″-hydroxy-dihydrofurano[1″,3″: 4′,5′] flavanone **(18)**	Flavanone	SB	Methanol	UV, HPLC, NMR, HMQC, HMBC	PTP 1B inhibitory activity	No activity	[135]
(2S)-5,7-Dihydroxy-3′-methoxy-2″*ξ*-(4″-hydroxyisopropyl) dihydrofurano[1″,3″:4′, 5′]flavanone **(19)**	Flavanone	SB	Methanol	UV, HPLC, NMR, HMQC, HMBC	PTP 1B inhibitory activity	No activity	[135]
(2S)-5,7,3′-Trihydroxy-2″*ξ*-(4″-hydroxyisopropyl) dihydrofurano[1″,3″ : 4′,5′] flavanone **(20)**	Flavanone	SB	Methanol	UV, HPLC, NMR, HMQC, HMBC	PTP 1B inhibitory activity	No activity	[135]
(2S)-5,7,3′-Trihydroxy-2″*ξ*-(4″-hydroxyisopropyl)-3″-hydroxy-dihydrofurano[1″,3″:4′,5′] flavanone **(21)**	Flavanone	SB	Methanol	UV, HPLC, NMR, HMQC, HMBC	PTP 1B inhibitory activity	No activity	[135]
Erythrabyssin I (**22**)	Pterocarpan	Rt	Methanol	UV, NMR, HPLC	Antimicrobial activity	Moderate antiyeast and antifungal activities	[136]
Erylatissin C (**23**)	Flavanone	SB	Methanol	UV, HPLC, NMR, HMQC, HMBC	PTP 1B inhibitory activity	No activity	[135]
Abyssinin III **(24)**	Flavanone	SB	Methanol	HPLC, NMR, HREI-MS, HMQC, HMBC, NOESY	Not tested	Not applicable	[82]
Indicanine B **(25)**	Coumarin	RB	DCM: MeOH	FTIR, UV, EI-MS, NMR	Antimicrobial activity	Active	[137]
Indicanine C **(26)**	Isoflavone	RB	DCM: MeOH	FTIR, UV, EI-MS, NMR	Antimicrobial activity	Not active	[137]
Cajanin **(27)**	Isoflavone	RB	DCM: MeOH	FTIR, UV, EI-MS, NMR	Antimicrobial activity	Not active	[137]
Abyssinone A **(28)**	Chalcone	SB	Methanol	UV, CD, NMR, HRMS	Not tested	Not applicable	[138]
Abyssinone B **(29)**	Chalcone	SB	Methanol	UV, CD, NMR, HRMS	Not tested	Not applicable	[138]
Abyssinone C **(30)**	Chalcone	SB	Methanol	UV, CD, NMR, HRMS	Not tested	Not applicable	[138]
Abyssinone D **(31)**	Chalcone	SB	Methanol	UV, CD, NMR, HRMS	Not tested	Not applicable	[138]
3-Methylbutein **(32)**	Chalcone	Rt	Chloroform: methanol (1 : 1)	UV, NMR, EI-MS, HMBC	Antimicrobial and antioxidant activities	Good bioactivities	[134]
2(S)-5,5′,7-Trihydroxy-2′-prenyl-(2″,2″-dimethylpyrano)-(5″,6”:3′,4′)flavanone **(33)**	Flavanone	SB	Methanol	UV, CD, NMR, HRMS	PTP 1B inhibitory activity	Good activity	[138]
i2(S)-5,5′,7-Trihydroxy-[2”-(5″- hydroxy)-methylpyrano]-(5″,6”:3′,4′)flavanone **(34)**	Flavanone	SB	Methanol	UV, CD, NMR, HRMS	PTP 1B inhibitory activity	No activity	[138]
2(S)-5,7-Dihydroxy-3′-methoxy-[2”-(5″-hydroxy)-methylpyrano]-(5″,6”:3′,4′)flavanone **(35)**	Flavanone	SB	Methanol	UV, CD, NMR, HRMS	PTP 1B inhibitory activity	Good activity	[138]
2(S)-5,7-Dihydroxy-[(5″,6”:3′,4′)-(2″,2″-dimethylpyrano)-(5‴,6‴:5′,6′)]-(2‴,2‴-dimethylpyrano)flavanone **(36)**	Flavanone	SB	Methanol	UV, CD, NMR, HRMS	PTP 1B inhibitory activity	No activity	[138]
2(S)-5,7-Dihydroxy-5′-prenyl-[2″,2”-(3″-hydroxy)-dimethylpyrano]-(5″,6”:3′,4′)flavanone **(37)**	Flavanone	SB	Methanol	UV, CD, NMR, HRMS	PTP 1B inhibitory activity	Good activity	[138]
2(S)-5,7-Dihydroxy-5′-methoxy-[2″,2”-(3″-hydroxy)-dimethyl-pyrano]-(5″,6”:3′,4′)flavanone **(38)**	Flavanone	SB	Methanol	UV, CD, NMR, HRMS	PTP 1B inhibitory activity	Good activity	[138]
2(S)-5,7-Dihydroxy- [2″,2”-(3″,4″-dihydroxy)-dimethylpyrano]-(5″,6”:3′,4′)flavanone **(39)**	Flavanone	SB	Methanol	UV, CD, NMR, HRMS	PTP 1B inhibitory activity	No activity	[138]
2(S)-5,7-Dihydroxy-5′-prenyl-[2″,2”-(3″,4″-dihydroxy)-dimethylpyrano)]-(5″,6”:3′,4′)flavanone **(40)**	Flavanone	SB	Methanol	UV, CD, NMR, HRMS	PTP 1B inhibitory activity	Good activity	[138]
2(S)-5,6′,7-Trihydroxy-5′-prenyl-[2″,2”-(3″,4″-dihydroxy)-dimethylpyrano]-(5″,6”:3′,4′)flavanone **(41)**	Flavanone	SB	Methanol	UV, CD, NMR, HRMS	PTP 1B inhibitory activity	Good activity	[138]
2(S)-5,5′,7-Trihydroxy-[2″,2”-(4″-chromanone)-dimethylpyrano]-(5″,6”:3′,4′)flavanone **(42)**	Flavanone	SB	Methanol	UV, CD, NMR, HRMS	PTP 1B inhibitory activity	No activity	[138]
2(S)-5′,7-Dihydroxy-[2″,2”-(3″-hydroxy)-dimethylpyrano]-(5″,6”:3′,4′)flavanone **(43)**	Flavanone	SB	Methanol	UV, CD, NMR, HRMS	PTP 1B inhibitory activity	No activity	[138]
2(S)-5′,7-Dihydroxy-[2″,2”-(3″,4″-dihydroxy)-dimethylpyrano]-(5″,6”:3′,4′)flavanone **(44)**	Flavanone	SB	Methanol	UV, CD, NMR, HRMS	PTP 1B inhibitory activity	No activity	[138]
Abyssinin I **(45)**	Flavanone	SB	Methanol	HPLC, NMR, HREI-MS, HMQC, HMBC, NOESY	Not tested	Not applicable	[82]
Abyssinin II **(46)**	Flavanone	SB	Methanol	HPLC, NMR, HREI-MS, HMQC, HMBC, NOESY	Not tested	Not applicable	[82]
Licochalcone A **(47)**	Chalcone	Rt	Chloroform: methanol (1 : 1)	UV, NMR, EI-MS, HMBC	Antimicrobial and antioxidant activities	Weak activity	[134]
Abyssinone V 4′-methyl ether **(48)**	Flavanone	SB	Methanol	UV, HPLC HREIMS, NMR, HMQC, HMBC, NOESY	Not tested	Not applicable	[82]
Abyssinoflavanone IV **(49)**	Prenylated flavanone	SB	Methanol	UV, NMR, CD, HREI-MS, HPLC, HMQC, HMBC, NOESY	Not tested	Not applicable	[82, 138]
Abyssinoflavanone V **(50)**	Prenylated flavanone	SB	Methanol	UV, NMR, CD, HREI-MS, HPLC, HMQC, HMBC, NOESY	Not tested	Not applicable	[82, 138, 139]
Abyssinoflavanone VI **(51)**	Prenylated flavanone	SB	Methanol	UV, NMR, CD, HREI-MS, HPLC, HMQC, HMBC, NOESY	Not tested	Not applicable	[82, 138–140]
Sigmoidin D **(52)**	Flavanone	Rt, SB	Chloroform: methanol (1 : 1), methanol	UV, NMR, CD, EI-MS, HRMS, HMBC	Antimicrobial and antioxidant activities, PTP 1B inhibitory activity	Weak antimicrobial and antioxidant activities, no activity	[82, 134, 138]
5,7-Dihydroxy-2′,4′,5′-trimethoxyisoflavanone **(53)**	Isoflavanone	Rt	Chloroform: methanol (1 : 1)	UV, NMR, EI-MS, HMBC	Antimicrobial and antioxidant activities	Weak activity	[134]
5-Prenylpratensein **(54)**	Isoflavone	Rt	Chloroform: methanol (1 : 1)	UV, NMR, EI-MS, HMBC	Antimicrobial and antioxidant activities	Weak activity	[134]
Abyssinone I **(55)**	Flavanone	RB	80% aqueous MeOH, ether	UV, HPLC	Antimicrobial activity	Moderate activity	[136, 139, 141]
Abyssinone II **(56)**	Flavanone	RB	80% aqueous MeOH	UV, HPLC	Antimicrobial and PTP1B inhibitory activities	Moderate and no activity	[136, 141]
			Ether				
			Ethyl acetate				
Abyssinone III **(57)**	Flavanone	RB	Ethyl acetate	HPLC, IR, UV, MS, CD, NMR	PTP1B inhibitory and antifungal activities	Weak activity	[136, 142]
			Ether				
Abyssinone IV **(58)**	Flavanone	RB	80% aqueous MeOH	UV, NMR, HMBC, EI-MS, HPLC	Antimicrobial and antioxidant activities	Moderate activity	[134, 136, 141]
			Chloroform : methanol (1 : 1)				
Abyssinone V **(59)**	Flavanone	Rt, SB	Chloroform : methanol (1 : 1)	UV, NMR, HMBC, HREI-MS, CD, HPLC, NOESY	Antimicrobial, antiplasmodial, antioxidant. and PTP1B inhibitory activities	Weak activity	[82, 134, 136, 141–143]
			Methanol				
			Ether				
			Ethyl acetate				
Abyssinone VI **(60)**	Isoflavone	NS	Ether	UV, HPLC	Antifungal activity	Not reported	[136]
Abyssinone VII **(61)**	Chalcone	Rt	Chloroform : methanol (1 : 1), ether	UV, NMR, EI-MS, HMBC, HPLC	Antimicrobial and antioxidant activities	Good activity	[134, 136]
Erythribyssin N **(62)**	Benzofuran	SB	Methanol	HPLC, IR, UV, MS, NMR	AMPK activity	Marked stimulation	[144]
Sigmoidin K **(63)**	Benzofuran	SB	Methanol	HPLC, IR, UV, MS, NMR	AMPK activity	Marked stimulation	[144]
Isosojagol **(64)**	Benzofuran	SB	Methanol	HPLC, IR, UV, MS, NMR	AMPK activity	Less stimulation	[144]
Erythribyssin F **(65)**	Coumestan	SB	Methanol	HPLC, IR, UV, MS, NMR	AMPK activity	Marked stimulation	[144]
Eryvarin Q **(66)**	Coumestan	SB	Methanol	HPLC, IR, UV, MS, NMR	AMPK activity	Less stimulation	[144]
Erypoegin F **(67)**	Coumestan	SB	Methanol	HPLC, IR, UV, MS, NMR	AMPK activity	Marked stimulation	[144]
Erythribyssin H **(68)**	Benzofuran	SB	Methanol	HPLC, IR, UV, MS, NMR	AMPK activity	Less stimulation	[144]
Eryvarin R **(69)**	Benzofuran	SB	Methanol	HPLC, IR, UV, MS, NMR	AMPK activity	Less stimulation	[144]
Erythribyssin E **(70)**	Isoflavanone	RB	Ethyl acetate	IR, UV, MS, CD, NMR	PTP 1B inhibitory activity	Strong activity	[142]
Prostratol C **(71)**	Isoflavanone	RB	Ethyl acetate	IR, UV, MS, CD, NMR	PTP 1B inhibitory activity	Strong activity	[142]
Erythribyssin J **(72)**	Isoflavanone	RB	Ethyl acetate	IR, UV, MS, CD, NMR	PTP 1B inhibitory activity	Strong activity	[142]
5-Deoxyabyssinin II **(73)**	Flavanone	RB	Ethyl acetate	IR, UV, MS, CD, NMR	PTP 1B inhibitory activity	Strong activity	[142]
7-Demethylrobustigenin **(74)**	Isoflavone	Rt	Chloroform : methanol (1 : 1)	UV, NMR, EI-MS, HMBC	Antimicrobial and antioxidant activities	Weak activity	[134]
Erythribyssin K **(75)**	Flavanone	RB	Ethyl acetate	IR, UV, MS, CD, NMR	PTP 1B inhibitory activity	No activity	[142]
Erythrabyssin II (**76**)	Pterocarpan	Rt	Chloroform : methanol (1 : 1), methanol	UV, NMR, HPLC	Antimicrobial (antibacterial) and radical scavenging properties	Good radical scavenging, antiyeast and antifungal activities	[134, 136]
Liquiritigenin **(77)**	Flavanone	RB	Ethyl acetate	IR, UV, MS, CD, NMR	PTP 1B inhibitory activity	No activity	[142]
Liquiritigenin-50-O-methyl ether **(78)**	Flavanone	RB	Ethyl acetate	IR, UV, MS, CD, NMR	PTP 1B inhibitory activity	No activity	[142]
Burttinone **(79)**	Flavone	SB	Methanol	UV, NMR, CD, HRMS	PTP 1B inhibitory activity	Good activity	[138]
Burttinonedehydrate **(80)**	Flavone	SB	Methanol	UV, NMR, CD, HRMS	PTP 1B inhibitory activity	Good activity	[138]
Erythribyssin G **(81)**	Flavanone	RB	Ethyl acetate	IR, UV, MS, CD, NMR	PTP 1B inhibitory activity	Weak activity	[142]
Erythribyssin I **(82)**	Flavanone	RB	Ethyl acetate	IR, UV, MS, CD, NMR	PTP 1B inhibitory activity	No activity	[142]
7-Hydroxy-4′-methoxy-3-prenylisoflavone **(83)**	Isoflavone	SB	Methanol	UV, FTIR, TLC, NMR, HMBC	Antimicrobial and antiplasmodial activities	Moderately active	[145]
Octacosyl-E-ferulate (erythrinasinate A) **(84)**	Coumaric acid	Rt	Chloroform : methanol (1 : 1)	UV, NMR, EI-MS, HMBC	Antimicrobial and antioxidant activities	Weak activity	[134,145]
Erythrabyssin I **(85)**	Pterocarpan	NS	Ether, 80% MeOH	UV, NMR, EI-MS, HMBC, HPLC	Antifungal activity	Good activity	[134, 136, 141]
Erythrabyssin II **(86)**	Pterocarpan	Rt	Chloroform : MeOH (1 : 1), 80% MeOH	UV, NMR, EI-MS, HMBC, HPLC	Antimicrobial and antioxidant activities	Moderate activity	[134, 136, 141]
Genistein **(87)**	Isoflavone	Rt, Tw	Chloroform : methanol (1 : 1)	UV, NMR, EI-MS, HMBC	Antimicrobial and antioxidant activities	Weak activity	[134]
Neobavaisoflavone **(88)**	Flavanone	Rt	Chloroform : methanol (1 : 1)	UV, NMR, EI-MS, HMBC	Antimicrobial and antioxidant activities	Weak activity	[134]
Semilicoisoflavone B **(89)**	Isoflavone	Rt	Chloroform : methanol (1 : 1)	UV, NMR, EI-MS, HMBC	Antimicrobial and antioxidant activities	Weak activity	[134]
Sigmoidin A **(90)**	Flavanone	SB	Methanol	UV, HPLC HREI-MS, HMQC, HMBC, NOESY NMR	Antilipase activity	Exhibited antilipase activity	[82, 146]
Sigmoidin B **(91)**	Flavanone	Rt	Chloroform : methanol (1 : 1)	UV, NMR, HREI-MS, HMBC, NOESY	Antimicrobial and antioxidant activities	Good activities	[82, 134]
Sigmoidin B 4'-(methyl ether) **(92)**	Flavanone	SB	Methanol	UV, HPLC HREI-MS, HMQC, HMBC, NOESY NMR	Not tested	Not applicable	[82]
Phaseolin **(93)**	Chalcone	NS	Ether	UV, NMR, HMBC, EI-MS, HPLC	Antimicrobial activity	Good activity (antiyeast and antifungal activities)	[134, 136, 141]
Phaseollidin **(94)**	Chalcone	Rt	Chloroform : methanol (1 : 1), ether	UV, NMR, HMBC, EI-MS, HPLC	Antimicrobial and antioxidant activities	Weak activity	[134, 136]
Erythrartine/11-methoxyerysodine (**95**)	Alkaloid	Sd	Chloroform	TLC, MS, UV, NMR,	Anti-HIV-1 and cytotoxicity	Weak activity	[59]
Caryolane-1,9-diol (**96**)	Sesquiterpene	Rt	Chloroform : methanol (1 : 1)	UV, NMR, HMBC, EI-MS, HPLC	Antimicrobial and antioxidant activities	Weak activity	[134]
Abyssaponin A **(97)**	Triterpenoid	SB	Ethanol	NMR, UV, HRESI-TOFMS, ESI-MS/MS	Anticancer activity	Strong activity	[147]
Abyssaponin B **(98)**	Triterpenoid	SB	Ethanol	NMR, UV, HRESI-TOFMS, ESI-MS/MS	Anticancer activity	Strong activity	[147]
Soyasapogenol B **(99)**	Triterpenoid	SB	Ethanol	NMR, UV, HRESI-TOFMS, ESI-MS/MS	Anticancer activity	Strong activity	[147]
Abyssinoside A **(100)**	Flavanone	SB	Ethanol	NMR, UV, HRESI-TOFMS, ESI-MS/MS	Anticancer activity	Moderate activity	[147]
Abyssinoside B **(101)**	Flavanone	SB	Ethanol	NMR, UV, HRESI-TOFMS, ESI-MS/MS	Anticancer activity	Moderate activity	[147]
Abyssinoside C **(102)**	Flavanone	SB	Ethanol	NMR, UV, HRESI-TOFMS, ESI-MS/MS	Anticancer activity	Weak activity	[147]
Abyssinoside D **(103)**	Flavanone	SB	Ethanol	NMR, UV, HRESI-TOFMS, ESI-MS/MS	Anticancer activity	Moderate activity	[147]
Schaftoside **(104)**	Flavanone	SB	Ethanol	NMR, UV, HRESI-TOFMS, ESI-MS/MS	Not tested	Not applicable	[147]
Isoschaftoside **(105)**	Flavanone	SB	Ethanol	NMR, UV, HRESI-TOFMS, ESI-MS/MS	Not tested	Not applicable	[147]
Vicenin-2 **(106)**	Flavanone	SB	Ethanol	NMR, UV, HRESI-TOFMS, ESI-MS/MS	Not tested	Not applicable	[147]
Hovetrichoside C **(107)**	Aurananol	SB	Ethanol	NMR, UV, HRESI-TOFMS, ESI-MS/MS	Not tested	Not applicable	[147]
Sigmoidin C **(108)**	Flavanone	Rt	Chloroform : methanol (1 : 1), methanol	UV, NMR, HREI-MS, HMBC, NOESY, HPLC	Antimicrobial and antioxidant activities	Weak activity	[82,134]
Sigmoidin F **(109)**	Flavanone	Rt, SB	Chloroform : methanol (1 : 1), methanol	UV, NMR, HREI-MS, HMBC, HPLC, NOESY	Antimicrobial and antioxidant activities	Weak activity	[82, 134]
Quercetin **(110)**	Flavone	RB	Acetone	HRMS, NMR, HMBC	Radical scavenging properties	Moderately active	[133]
5,4′-di-*O*-Methylalpinumisoflavone **(111)**	Isoflavone	RB	DCM: MeOH	FTIR, UV, EI-MS, NMR	Antimicrobial activity	Not active	[137]
Erycristagallin **(112)**	Pterocarpan	RB	Acetone	HRMS, NMR, HMBC	Radical scavenging properties	Moderately active	[133]
Shinpterocarpin **(113)**	Pterocarpan	RB	Acetone	HRMS, NMR, HMBC	Radical scavenging properties	Moderately active	[133]
Sandwicensin **(114)**	Pterocarpan	Rt	Chloroform : methanol (1 : 1)	UV, NMR, EI-MS, HMBC	Antimicrobial and antioxidant activities	Weak activity	[134]
3,6-Caryolanediol **(115)**	Sesquiterpenes	Rt	Chloroform : methanol (1 : 1)	UV, NMR, EI-MS, HMBC	Antimicrobial and antioxidant activities	Weak activity	[134]
Clovane-2,9-diol **(116)**	Sesquiterpenes	Rt	Chloroform : methanol (1 : 1)	UV, NMR, EI-MS, HMBC	Antimicrobial and antioxidant activities	Weak activity	[134]
7-Hydroxy-2-[4-methoxy-3-(3-methylbut-2-enyl) phenyl] chroman-4-one **(117)**	Flavanone	RB	Ethyl acetate	IR, UV, MS, CD, NMR	PTP 1B inhibitory activity	Strong activity	[142]
Sigmoidin E **(118)**	Flavanone	Rt, SB	Chloroform : methanol (1 : 1), methanol	UV, NMR, CD, HREI-MS, HPLC, HMQC, HMBC, NOESY	Antimicrobial, antioxidant and PTP 1B inhibitory activities	Weak antimicrobial and antioxidant activities, no activity	[82, 134, 138, 139]
Sigmoidin G **(119)**	Flavanone	SB	Methanol	UV, CD, NMR, HRMS	PTP 1B inhibitory activity	No activity	[138, 139]
9-Ethyldodecyl-4-methoxybenzoate (**120**)	Benzoate ester	SB	Methanol	TLC, NMR	Antibacterial and termicidal activity	Moderate antibacterial activity	[62]
Lupinifolin (**121**)	Flavonoid	SB	Methanol	TLC, NMR	Antibacterial and termicidal activity	Moderate antibacterial activity	[62]
Kaempferol 3-O-(2-O-b-D-glucopyranosyl-6-O-a-L-rhamnopyranosyl-b-D-glucopyranoside (**122**	Flavonol	Fl	Methanol (acidified)	NMR, DQF-COSY	Not tested	Not applicable	[148]

NS: not specified; Fl: flowers, Sd: seeds; SB: stem bark, Rt: roots; RB: root bark; Tw: twig; FTIR: Fourier transform infrared spectroscopy; ESI-MS/MS: electron spray ionization tandem mass spectrometry; HRESI-TOFMS: high-resolution electron spray ionization time-of-flight mass spectrometry; HMBC: heteronuclear multiple bond correlation spectroscopy; HMQC: heteronuclear multiple quantum coherence spectroscopy; CD: circular dichroism spectroscopy; HRMS: high-resolution mass spectrometry; NOESY: nuclear overhauser effect spectroscopy; DQF-COSY: double quantum filtered correlation spectroscopy; UV: ultraviolet-visible spectroscopy; MS: mass spectrometry; NMR: nuclear magnetic resonance; TLC: thin-layer chromatography; AMPK: adenosine monophosphate-activated protein kinase.

**Table 5:** Pharmacological profile of different parts of *E. abyssinica*.

Activity	Model used	Plant part	Extract/compound	Bioassay	Results	Author(s)
Antioxidant	*In vitro*	Ethanol, methanol	Leaves, root bark	DPPH radical scavenging assay	Extract showed dose-dependent DPPH radical scavenging activity that was comparable to that of ascorbic acid at all doses (10–320 *μ*g/mL)	[127,159]
Antioxidant	*In vitro*	Abyssinone VII Sigmoidin B Eryvarin L 3-Methylbutein	Stem bark	DPPH radical scavenging assay	After 1 h, the DPPH radical scavenging activity was as follows: abyssinone VII: IC_50_ = 25 *μ*g/mL, sigmoidin B: IC_50_ = 18.5 *μ*g/mL, eryvarin L: IC_50_ = 29 *μ*g/mL, and 3-methylbutein: IC_50_ = 37 *μ*g/mL, ascorbic acid: IC_50_ = 18* μ*g/mL, gallic acid: IC_50_ = 4 *μ*g/mL. and quercetin: IC_50_ = 7 *μ*g/mL	[134]
Antioxidant	*In vitro*	Acetone	Root bark	DPPH radical scavenging assay	After 30 minutes, the DPPH radical scavenging activity was as follows: crude extract: IC_50_ = 7.7 *μ*M, abyssinone IV: 32.4 *μ*M, abyssinone V: 30.1 *μ*M, abyssinin III: 21.7 *μ*M, erycristagallin: IC_50_ = 8.2 *μ*M, 3-hydroxy-9-methoxy-10-(3,3-dimethylallyl) pterocarpene: IC_50_ = 10.8 *μ*M, and quercetin: IC_50_ = 5.4 *μ*M	[133]
Anti-inflammatory	*In vivo*	Root bark	Methanol	Chronic trypanosomiasis-induced neuroinflammation mouse model	The aqueous extract-treated group (50 mg/kg) had lower astrocyte reactivity (34,545 astrocytes/mm^3^) than the untreated group (69,886 astrocytes/mm^3^). Also, they had a reduced degree of neuroinflammation (1.2) compared to the untreated group (2.8). The extract was thought to reduce the infiltration of inflammatory cells into the cerebrum.	[50]
			Water		The methanol extract did not have a significant effect on the modulation of neuroinflammation	
Antihyperglycemic	*In vivo*	Root bark	Water	Oral glucose tolerance assay using male guinea pigs (*Cavia porcellus*)	38% inhibition factor against hyperglycemia at a dose of 500 mg/kg (6 mg/kg glibenclamide = 49.6%)	[114]
Antihyperglycemic	*In vivo*	Leaf	Ethanol	Oral glucose tolerance assay using male	After 4 hours of hyperglycemia induction, the extract significantly and dose dependently reduced the mean blood glucose; 100 mg/kg = 25%, 200 mg/kg = 46.4%, 400 mg/kg = 60.7%, and 5 mg/kg glibenclamide = 35.7%	[159]
				Wistar albino rats		
Anticancer	*In vitro*	Stem bark	Ethanol	MTT and protein tyrosine phosphatase (PTP1B) inhibitory assay	Compounds exhibited PTP1B inhibitory activity with IC_50_ values ranging from 4.2 to 19.3 *μ*M and strong cytotoxic activity with IC_50_ values from 5.6 to 28.0 *μ*M	[160]
					After 72 hours of exposure; MCF7: IC_50_ = 19.4 *μ*M, MCF/AMR: IC_50_ = 12.0 *μ*M, MCF/ADR: IC_50_ = 16.1 *μ*M, MDA-MB-231: IC_50_ = 28.0 *μ*M, and PTB1B: IC_50_ = < 30 *μ*M.	
Anticancer	*In vitro*	Seeds	Chloroform	Sulforhodamine B assay using HeLa, Hep-G2, HEP-2, HCT116, MCF-7, and HFB4 cells	The crude alkaloidal fraction showed cytotoxic activity against the tumor cells with IC_50_ values of 13.8, 10.1, 8.16, 13.9, 11.4, and 12.2 *μ*g/mL against HeLa, Hep-G2, HEP-2, HCT116, MCF-7, and HFB4 cells, respectively.	[59]
					After 72 hours of exposure, the IC_50_ of isolated compounds on Hep-G2 and HEP-2 cells were as follows, respectively: erythraline: IC_50_ = 21.60 and 15.8 *μ*g/mL, erysodine: IC_50_ = 19.90 and 11.8 *μ*g/mL	
					Erysotrine: IC_50_ = 21.60 and 15.8 *μ*g/mL, 8-oxoerythraline: IC_50_ = 18.50 and 3.89 *μ*g/mL, 11-methoxyerysodine: IC_50_ = 11.50 and 11.4 *μ*g/mL	
Antianaemic	*In vivo*	Stem bark	Water extract	Phenyl hydrazine anaemia-induced mouse model	Improved haematinic activity in a dose-dependent manner. Extracts increased the red blood cell differentials in anaemic rats at increasing doses of 50, 100, and 350 mg/kg	[128]
Antiobesity	*In vitro*	Stem bark	Sigmoidin A	Pancreatic lipase and alpha-glucosidase inhibition assay	IC_50_ = 4.5 and 62.5 *μ*M for pancreatic lipase and alpha glucosidase inhibition, respectively (orlistat = 0.3 *μ*M, acarbose = 190.6 *μ*M)	[146]
Antipyretic and estrogenomimetic	*In vivo*	Stem bark	Methanol	Smart button data loggers using ovariectomized rats using	At a dose of 200 mg/kg, the extract reduced the average number of hot flushes (171 in treated vs. 264 in the untreated group). The treated group also had shorter durations of hot flushes (683 minutes) compared to the untreated (1935 minutes)	[161]
Hepatoprotective	*In vivo*	Stem bark	Water	Nonalcoholic fatty liver disease model using rats to evaluate the fasting blood glucose, insulin tolerance, hepatic triglycerides, serum biochemistry, and liver histology	The extract had significant effects on fasting blood glucose as well as hepatic indices including liver weights, hepatic triglycerides, liver weight-body weight ratio, serum AST, serum ALT levels, serum triglycerides, serum total cholesterol, and serum LDL-cholesterol; however, the extracts showed no significant effects on HDL-cholesterol. At high doses (400 mg/kg), the extract protected the liver against inflammation, steatosis, and hepatic ballooning	[162]
Wound healing	*In vivo*	Leaf and stem bark	Methanol	Wound incision assay	82.1 and 88.7% wound area healed after 15 days for the stem bark and leaf extract, respectively, at a dose of 10% w/w	[94]
					The mean skin protein was 32.5 and 35.5% for the stem bark and leaf, respectively (oxytetracycline = 40.5%).	
					Although the leaf extract had better healing properties than the bark, there was no significant difference between both extracts and the negative control	
Antiplasmodial	*In vivo*	Stem and root bark	Acetone	4-day ANKA suppressive bioassay using *P. berghei*	% chemosuppression: root (49.7%), stem (44.2%), and chloroquine (97.3%)	[163]
Antiplasmodial	*In vitro*	Leaves	n-Hexane	Nonradioactive antiplasmodial 3H hypoxanthine inhibition assay using *P. falciparum* multidrug-resistant Indochicha I (W2) and chloroquine-sensitive Sierra Leone I (D6)	After 24 hours, n-hexane extract: IC_50_ = 0.0 *μ*g/mL, DCM extract: IC_50_ = 190.1 *μ*g/mL, methanol extract: IC_50_ = 348.2 *μ*g/mL, mefloquine: IC_50_ = 19.2 *μ*g/mL.	[145]
			Dichloromethane (DCM)			
			Methanol			
Antiplasmodial	*In vitro*	Stem	Ethyl acetate extract	Nonradioactive antiplasmodial 3H hypoxanthine inhibition assay using *P. falciparum* multidrug-resistant Indochicha I (W2) and chloroquine-sensitive Sierra Leone I (D6)	After 24 hours, ethyl acetate extract: D6: IC_50_ = 7.9 *μ*g/mL, W2: IC_50_ = 5.3 *μ*g/mL, chalcones: IC_50_ ranged from 10 to 16 *μ*M, flavanones: IC_50_ ranged from 4.9 to 13.6 *μ*M, isoflavonoids: IC_50_ ranged from 18.2 to 24.9 *μ*M, chloroquine: IC_50_ ranged from 0.009 to 0.08 *μ*M, and quinine: IC_50_ ranged from 0.04 to 0.21 *μ*M	[49]
			Isolated compounds (chalcones, flavanones, isoflavonoids)			
Antiplasmodial	*In vivo*	Stem and root bark	Methanol	Four-day ANKA suppressive bioassay using *P. berghei* and *P. falciparum*	At 50 mg/kg of the extract, % chemosuppression: root bark (77%), stem bark (32%), and 10 mg/kg chloroquine (6%). Survival time in extract-treated and chloroquine-treated groups was 2- to 3-fold higher than the untreated. For *P. falciparum,* IC_50_ of 7.81 *μ*g/mL (K1 strain)	[28, 164]
Antiviral	*In vitro*	Seeds and stem	Chloroform, ethanol	MTT assay using HIV-1-infected MT-4 cells	Stem alkaloidal fraction: IC_50_ = 53 *μ*M, efavirenz: IC_50_ = 45 *μ*M	[59, 112]
					Stem had antiviral activity (reduction factors of the viral titer of 10^4^) against polio, Semliki forest, and herpes viruses	
Antimycobacterial	*In vitro*	Stem bark	Methanol	Microdilution assay against *Mycobacterium tuberculosis, Mycobacterium kansasii, Mycobacterium fortuitum,* and *Mycobacterium smegmatis*	At a dose of 2 mg/mL, the extract completely inhibited the growth of all Mycobacterial strains (0 GU). However, at 1 mg/mL, there was significant growth of *Mycobacterium tuberculosis* (19741 GU), *Mycobacterium kansasii* (724 GU), *Mycobacterium fortuitum* (174 GU), and *Mycobacterium smegmatis* (4915 GU)	[165]
Antimycobacterial	*In vitro*	Root bark	Methanol	Microdilution assay against pan-sensitive strain (H37Rv), rifampicin-resistant strain (TMC-331), *Mycobacterium avium*	Antimycobacterial activity of extract against H37Rv: MIC = 0.39 mg/mL, TMC-331: MIC = 2.35 mg/mL, *Mycobacterium avium*: MIC = 0.39 mg/mL. The MICs of isoniazid were 0.25 *μ*g/mL and 9.38 *μ*g/mL for H37Rv and TMC-331, respectively	[126]
Antimycobacterial	*In vitro*	Stem bark	Methanol	Microdilution assay against *M. tuberculosis*	Percentage inhibition of colony formation of different combinations: 0.06 *μ*g/mL ethanol extract with 0.01 *μ*g/mL rifampicin and isoniazid = 99.2%, 0.06 *μ*g/mL methanol extract with 0.01 *μ*g/mL rifampicin and isoniazid = 99% and 0.01 *μ*g/mL rifampicin and isoniazid = 86.2%	[166]
			Ethanol			
Antihelmintic	*In vitro*	Stem bark	Ethanol	Worm motility assessment assay on *Ascaridia galli*	After 24 hours of exposure, at 50 mg/kg of extracts, average number of worms immobilized out of 10: leaf = 9.46, stem = 7.17, root = 7.92, piperazine = 10	[124]
		Root bark				
		Leaves				
Antihelmintic	*In vitro*	Leaves	Ethanol	Worm motility assessment assay on *Ascaridia galli*	At 5% concentration of extracts, average number of worms immobilized out of 10 at different times: 12 h = 5, 24 h = 6, 36 h = 9, 48 h = 10	[120]
Antibacterial	*In vitro*	Stem and root barks, whole plant, leaves	Ethanol, methanol, chloroform, water	Microbroth dilution assay against *S. aureus E. coli,*	Ethanolic extracts inactive against *E. coli, S. typhi,* and *P. aeruginosa*. Extracts exhibited different antibacterial activities against *S. aureus* depending on the part of the plant and also the location from where they were harvested. In Mbarara, the root extract was more active (MIC 31.3 mg/mL) than the stem extract (MIC = 3.5 mg/mL). On the other hand, the root extract of Bushenyi was more active (31.3 mg/mL) than that of Ntungamo (4.7 mg/mL).	[19, 26, 91, 123, 127]
				*S. typhi*, *Bacillus cereus,* and *P. aeruginosa*	Methanolic extract showed better antibacterial activity (6.0 mm inhibition diameter, MIC = 0.23 mg/mL) against *S. aureus* than the positive reference controls: ampicillin (4.0 mm) and amoxicillin (5.0 mm)	
				*In vitro* antidiarrheal activity	Chloroform extract of the whole plant had bioactivity against *S. aureus*, with 7.45 mm inhibition zone diameter	
					Methanolic extract of root bark showed bioactivity against *S. aureus*, *B. cereus,* and *P. aeruginosa* with MIC and MBC of 3.125, 50.00, and 125.00, and 6.25, 100.00, and 250.00 mg/mL, respectively. Aqueous extract of root bark showed bioactivity against *S. aureus*, *B. cereus, E. coli,* and *P. aeruginosa* with MIC and MBC of 3.125, 12.50, 250.00, and 125.00, and 3.125, 25.00, 250.00 and 250.00 mg/mL, respectively.	
					Leaf powder exhibited potential antidiarrheal activity in mice.	
Antibacterial	*In vitro*	Stem and root bark	Methanol	Microbroth dilution assay against *Bacillus cereus, E. coli, Micrococcus luteus,* and *P. aeruginosa*	The extracts were not active on all the bacterial strains	[100]
Antibacterial and antifungal	*In vitro*	Root bark	Erythrabyssins I and II	Microbroth dilution assay against *E. coli, S. aureus, Bacillus subtilis, Saccharomyces cerevisiae, Penicillium crustosum, P. aeruginosa, Candida utilis, Mucor mucedo, Cryptococcus neoformans,* and *Candida albicans*	*E. coli* and *P. aeruginosa*: MIC values of all compounds were greater than 100 *μ*g/mL;	[60, 141]
			Abyssinones I, II, III, IV, V, VI		*S. aureus*: with exception of abysssinone II and VI, all the other compounds had MIC values below 100 *μ*g/mL.	
			Phaseolin		*Bacillus subtilis:* with exception of abyssinones II and VI, all the other compounds had MIC values below 100 *μ*g/mL.	
			Phaseollidin, extract		*Penicillium crustosum:* MIC values of all compounds were greater than 100 *μ*g/mL.	
					*S. cerevisiae* and *C. utilis:* with exception of erythrabyssin I and phaseolin, all the other compounds had MIC values above 100 *μ*g/mL.	
					*M. mucedo:* with exception of erythrabyssin I, abyssinones I and II, Phaseolin, all the other compounds had MIC values greater than 100 *μ*g/mL.	
					Extract had effective MICs at 25% (w/v) and 12.5% (w/v) with moderate fungal growth observed at 6.25% (w/v) against *C. neoformans* and *C. albicans*	
Antibacterial and antifungal	*In vitro*	Stem bark	Hexane, dichloromethane, methanol	Microbroth dilution assay against *E. coli, S. aureus,* methicillin-resistant *S. aureus* (MRSA), *P. aeruginosa, Klebsiella pneumoniae, Microsporum gypseum, Trichophyton mentagrophytes, C. albicans, Cryptococcus neoformans*	Extracts not active on *E. coli,* weak activity against *P. aeruginosa* and *K. pneumoniae* (MIC greater than 50 mg/mL). The methanol extract more active on MRSA (MIC = 6.25 mg/mL) and DCM on *S. aureus* (MIC = 25.0 mg/mL). Hexane extracts were the least active on all strains.	[62, 167]
					All extracts had good activity against *M. gypseum* (MIC less than 12.5 mg/mL) but weak activity against *C. albicans* and *C. neoformans* (MIC greater than 100 mg/mL). The hexane extract was active on *T. mentagrophytes* (MIC = 25.0 mg/mL).	
					Lupinifolin and 9-ethyldodecyl 2-hydroxy-4-methoxybenzoate from methanolic extract had zone of inhibition of 9.0 mm each against *B. subtilis* and *E. coli*, respectively. The compounds and crude extract inhibited *Fusarium* spp., *Trichophyton* spp., and *Penicillium* spp. with inhibition zones of 9.0–18.0 mm.	

MIC: minimum inhibitory concentration; IC_50_: inhibitory concentration; GU: growth units.

## Data Availability

This is a review article and no raw experimental data were collected. All data generated or analyzed during this study are included in this published article.

## Abbreviations

AMPK: Adenosine monophosphate-activated protein kinase
CNS: Central nervous system
*E. abyssinica*: *Erythrina abyssinica* Lam. ex. DC.
HIV: Human immunodeficiency virus
LD_50_: Median lethal dose
MIC/IC_50_: Minimum inhibitory concentration
PLA2: Phospholipase A2
WHO: World Health Organization.
